# Drp1 regulates mitochondrial health and controls skeletal muscle mass through the Erk1/2-Nur77 pathway

**DOI:** 10.1126/sciadv.aec0795

**Published:** 2026-05-08

**Authors:** Alice M. Ma, Peter H. Tran, Nicole L. Yang, Jennifer Ngo, Hirotaka Iwasaki, Wenjuan Ren, Simone Livit, Linsey Stiles, Sarah Wang, Trinity Ho, Emma Y. Yim, Noelle Morrow, Morgan M. Johnson, Caroline Cleary, Kai Zou, Rachelle H. Crosbie, Yuwei Jiang, Orian S. Shirihai, Jonathan Wanagat, Sushil Mahata, James A. Wohlschlegel, Andrea L. Hevener, Zhenqi Zhou

**Affiliations:** ^1^ Division of Endocrinology, Diabetes and Hypertension, Department of Medicine, University of California, Los Angeles, Los Angeles, CA 90095, USA.; ^2^ Division of Geriatrics, Department of Medicine, University of California, Los Angeles, Los Angeles, CA 90095, USA.; ^3^ Department of Exercise and Health Sciences, University of Massachusetts Boston, Boston, MA 02125, USA.; ^4^ Department of Integrative Biology and Physiology, University of California, Los Angeles, Los Angeles, CA 90095, USA.; ^5^ Center for Duchenne Muscular Dystrophy, University of California, Los Angeles, Los Angeles, CA 90095, USA.; ^6^ Department of Physiology and Biophysics, College of Medicine, University of Illinois at Chicago, Chicago, IL 60612, USA.; ^7^ VA San Diego Healthcare System, San Diego, CA 92161, USA.; ^8^ Department of Medicine, University of California, San Diego, La Jolla, CA 92093, USA.; ^9^ Department of Biological Chemistry, University of California, Los Angeles, Los Angeles, CA 90095, USA.; ^10^ Department of Medicine and VA Greater Los Angeles Healthcare System GRECC, Los Angeles, CA 90073 USA.; ^11^ Iris Cantor–UCLA Women’s Health Research Center, Los Angeles, CA 90095, USA.; ^12^ Molecular Biology Institute, University of California, Los Angeles, Los Angeles, CA 90095, USA.

## Abstract

The maintenance of skeletal muscle mass relies on mitochondrial quality control, including balanced dynamics and mitophagy. Dynamin-related protein 1 (Drp1), a central mediator of mitochondrial fission, is essential for these processes, yet its role in muscle mass regulation remains incompletely defined. Here, we show that acute Drp1 deletion in the skeletal muscle increases Parkin-mediated mitochondrial degradation, reduces mitochondrial DNA (mtDNA) content, and leads to severe muscle atrophy. Although dual deletion of Drp1 and Parkin restores mtDNA content, muscle loss persists. Mechanistically, Drp1 loss impairs mitochondrial respiratory chain activity, suppressing extracellular signal–regulated kinase 1/2 (Erk1/2) signaling and down-regulating the nuclear receptor subfamily 4 group A member 1 (Nur77). Pharmacologic β2-adrenergic receptor activation with clenbuterol reactivated Erk1/2, restored Nur77 expression, and rescued muscle atrophy. These findings define a Drp1-Erk1/2-Nur77 signaling axis linking mitochondrial integrity to skeletal muscle mass and identify a potential therapeutic target for muscle degeneration in mitochondrial and metabolic diseases.

## Teaser

Drp1 regulates skeletal muscle mass through an Erk1/2-Nur77 pathway that offers a therapeutic target.

## INTRODUCTION

Skeletal muscle atrophy occurs not only as a natural consequence of aging but also as a pathological feature of various diseases, including diabetes, cancer cachexia, and certain neurodegenerative disorders (*1*, *2*). Mitochondria serve as central hubs for cellular energy metabolism, calcium homeostasis, and cholesterol biosynthesis, and their dynamic regulation is critical for maintaining skeletal muscle integrity and function (*3*, *4*).

Mitochondria are highly dynamic organelles that continuously undergo fusion and fission, processes essential for maintaining mitochondrial function, quality control, cellular energy homeostasis, and adaptation to physiological stress (*5*, *6*). Mitochondrial fusion is regulated by the mitofusin 1 (Mfn1) and mitofusin 2 (Mfn2), which are located on the outer mitochondrial membrane, as well as optic atrophy 1 (Opa1), which is located on the inner mitochondrial membrane (*7*). In contrast, mitochondrial fission is primarily mediated by Drp1 (encoded by *Dnm1l* gene), a cytosolic guanosine triphosphatase (GTPase) that translocates to the mitochondrial outer membrane upon activation (*8*). Drp1 interacts with adapter proteins including mitochondrial fission factor (Mff), mitochondrial fission 1 (Fis1), and mitochondrial dynamics protein 49/51 (MiD49/51), facilitating mitochondrial division (*9*). Dysregulation of mitochondrial dynamics has been implicated in a variety of pathological conditions, including neurodegenerative diseases (*10*), metabolic disorders (*11*), and muscle atrophy (*12*, *13*), highlighting the importance of these processes in maintaining cellular health.

To sustain a healthy mitochondrial network, Drp1 facilitates the segregation and removal of damaged mitochondria (*14*, *15*). Although global deletion of Drp1 is embryonically lethal, studies have shown that conditional loss and gain of Drp1 expression regulates muscle growth and mass (*16*–*18*). However, the precise mechanisms by which Drp1 maintains skeletal muscle function remain incompletely understood.

Our previous studies of conventional muscle-specific *Dnm1l* heterozygous knockout (mDrp1^HET^) mice revealed that Drp1 is critical for mitochondrial complex II assembly and lipid oxidation, thereby influencing systemic glucose homeostasis and affecting maximal running speed and muscle endurance capacity (*19*, *20*). However, we were only able to obtain heterozygous mice, suggesting that skeletal muscle Drp1 null mutation is embryonically lethal. To circumvent this limitation and prevent developmental compensation of gene deletion, we generated a tamoxifen (TM)–inducible skeletal muscle–specific *Dnm1l* knockout (miDrp1^KO^) mouse model, allowing us to examine the direct effects of Drp1 loss in the skeletal muscle of adult mice. Here, we show that acute Drp1 deletion promotes a progressive decline in mitochondrial DNA (mtDNA) content, accompanied by a distinct metabolic profile that differs from the previously described mDrp1^HET^ mice (*19*). The loss of Drp1 elevates Parkin RBR E3 ubiquitin protein ligase (Parkin)–mediated mitochondrial degradation, contributing to mtDNA depletion, impaired mitochondrial activity, suppression of extracellular signal–regulated kinase 1/2 (Erk1/2) signaling, and down-regulation of nuclear receptor subfamily 4 group A member 1 (Nur77), a key regulator of muscle mass (*21*, *22*). Our findings suggest a direct link between mitochondrial dynamics and the transcriptional regulation of pathways governing muscle composition. We establish Drp1 as a key regulator of skeletal muscle homeostasis and uncover molecular mechanisms that could be leveraged for therapeutic interventions aimed at preserving muscle function in the context of metabolic-related diseases and aging-related muscle degeneration.

## RESULTS

### Skeletal muscle–specific *Dnm1l* null mice develop progressive muscle atrophy

**Fig. 1.**
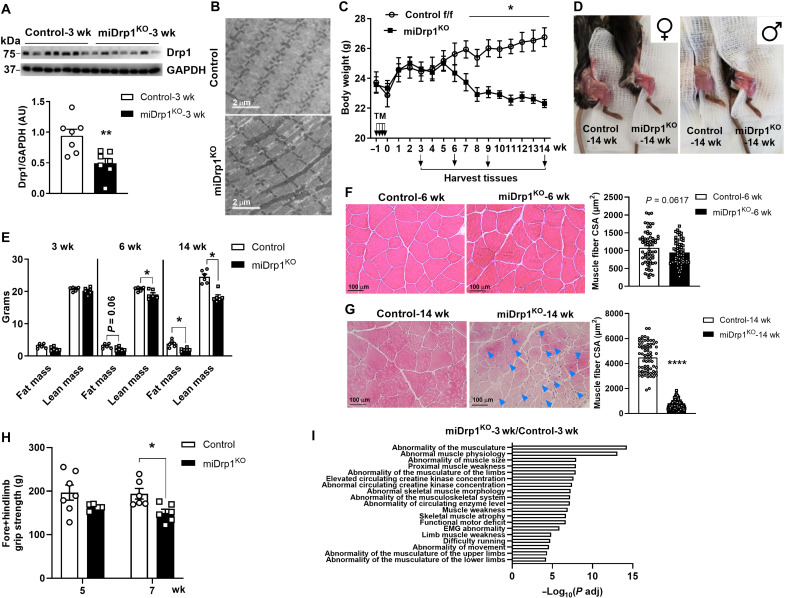
Acute skeletal muscle–specific *Dnm1l* deletion induces muscle wasting. (**A**) Western blot analysis validated the decreased Drp1 protein level in quadriceps muscles of miDrp1^KO^ mice at 3 weeks (*n* = 7) compared to controls (*n* = 7). The graph at the bottom represents the densitometry of Drp1 protein. wk, weeks; AU, arbitrary units; GAPDH, glyceraldehyde-3 phosphate dehydrogenase. (**B**) Electron microscopy images showing the elongated mitochondrial morphology in the soleus muscle of miDrp1^KO^ mice at 3 weeks post–*Dnm1l* deletion compared to control mice (scale bars, 2 μm). (**C**) Body weight of control (*n* = 6) and miDrp1^KO^ mice (*n* = 6). Tissues were harvested at 3, 6, 9, and 14 weeks post–*Dnm1l* deletion. (**D**) Picture of mouse muscles of both female and male control and miDrp1^KO^ mice at 14 weeks post–*Dnm1l* deletion. (**E**) Body composition of male control (*n* = 6) and miDrp1^KO^ mice (*n* = 6) at 3, 6, and 14 weeks post–*Dnm1l* deletion. H&E staining and muscle CSA (right) of gastrocnemius muscles from control and miDrp1^KO^ mice (**F**) at 6 weeks and (**G**) at 14 weeks after *Dnm1l* deletion (blue arrows indicate fibers with centralized nuclei; scale bars, 100 μm). Quantification of muscle CSA is shown on the right (*n* = 3). (**H**) Grip strength of control and miDrp1^KO^ mice at 5 weeks (control, *n* = 7; miDrp1^KO^, *n* = 6) and 7 weeks (control, *n* = 6; miDrp1^KO^, *n* = 7) post–*Dnm1l* deletion. (**I**) Human Phenotype Ontology enrichment analysis of RNA-seq data from quadriceps muscles of control and miDrp1^KO^ mice at 3 weeks. Data are presented as means ± SEM; unpaired Student’s *t* test two tailed. *P < 0.05; ****P < 0.0001.

Our previous study demonstrated that skeletal muscle expression of Drp1 is critical for survival, as homozygous *Dnm1l* gene deletion failed to produce a viable mouse (*19*). To overcome this limitation, we crossed floxed *Drp1* mice with a TM-inducible skeletal muscle–specific Cre mouse line (HSA-MerCreMer, under the control of the human skeletal actin, *ACTA1*, promoter) to generate a miDrp1^KO^ mouse model, which enables *Dnm1l* deletion in the skeletal muscle of adult mice, thereby avoiding confounding effects of development. Following 3 weeks of TM administration, Drp1 protein levels were reduced >50% in the skeletal muscle, whereas as expected, *Dnm1l* gene expression in the liver remained unchanged (Fig. 1A and fig. S1A). Consistent with the finding that Drp1 deletion impairs mitochondrial fission, we observed marked mitochondrial elongation in the skeletal muscle of miDrp1^KO^ mice (Fig. 1B and fig. S1B).

A notable reduction in body weight was observed in miDrp1^KO^ mice compared to controls, beginning 6 weeks after *Dnm1l* deletion and persisting for up to 14 weeks (Fig. 1C). This wasting phenotype was observed in both sexes of mice (Fig. 1D) and was primarily attributed to a decrease in lean mass and gastrocnemius muscle weight beginning at 6 weeks after *Dnm1l* deletion (Fig. 1E and fig. S1C). We found that reduction in muscle mass in miDrp1^KO^ mice was independent of TM dosage as even a single injection of TM promoted severe muscle atrophy 15 weeks after administration (fig. S1, D to F). Moreover, partial loss of *Dnm1l* is sufficient to disrupt muscle homeostasis as miDrp1^HET^ mice also exhibited a decline in body weight beginning 6 weeks after *Dnm1l* deletion (fig. S1G). Histological analysis showed a modest reduction in muscle cross-sectional area (CSA) at 6 weeks after *Dnm1l* deletion, progressing to a marked decrease accompanied by numerous centralized nuclei at 14 weeks (Fig. 1, F and G). Consistent with these structural changes and progressive muscle wasting, a significant reduction in grip strength for miDrp1^KO^ mice was observed at 7 weeks after *Dnm1l* deletion compared with control (Fig. 1H). Transcriptomic analysis by RNA sequencing (RNA-seq) revealed that numerous pathways related to muscle dysfunction were already enriched in miDrp1^KO^ skeletal muscle as early as 3 weeks post–*Dnm1l* deletion (Fig. 1I). Given the mechanistic complexity of muscle wasting mechanisms and the potential activation of compensatory pathways at later stages, we focused our attention on mice 3 weeks post–*Dnm1l* deletion, a time point before overt muscle atrophy.

### Distinct metabolic profiles in conventional versus inducible muscle-specific *Dnm1l* null mouse models

**Fig. 2.**
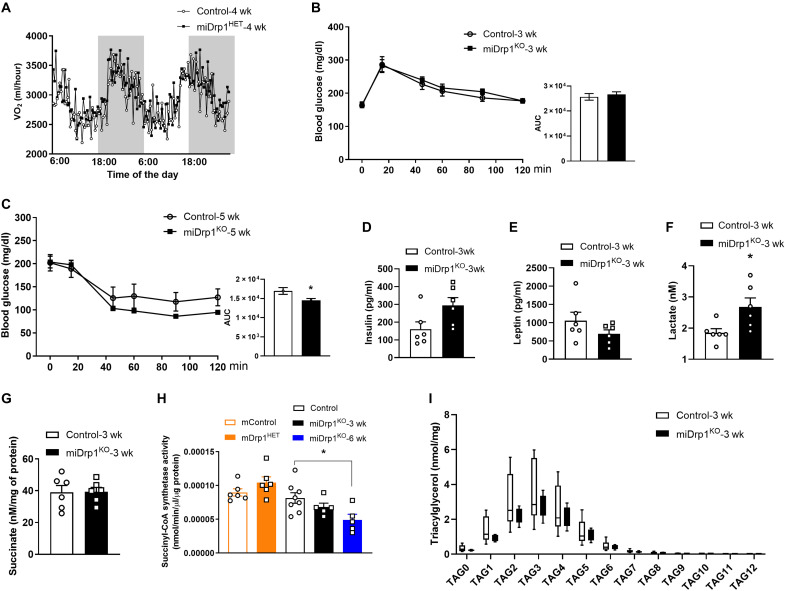
Comparative phenotypic analysis of conventional and inducible Drp1 deletion in the skeletal muscle. (**A**) Oxygen consumption rate of NC-fed control (*n* = 6) and miDrp1^HET^ (*n* = 6) mice at 4 weeks post–*Dnm1l* deletion. Open columns represent daytime, whereas shaded columns represent nighttime. The same cohort of mice was used for both GTT and ITT assays: (**B**) intraperitoneal GTT and AUC (area under the curve) for NC-fed control (*n* = 5) versus miDrp1^KO^ mice (*n* = 5) at 3 weeks post–*Dnm1l* deletion and (**C**) intraperitoneal ITT and AUC in NC-fed control (*n* = 5) versus miDrp1^KO^ mice (*n* = 5) at 5 weeks post–*Dnm1l* deletion. (**D**) Plasma insulin, (**E**) leptin, and (**F**) lactate levels in male miDrp1^KO^ (*n* = 6) versus control (*n* = 6) mice fed an NC diet at 3 weeks post–*Dnm1l* deletion. (**G**) Succinate levels and (**H**) succinyl-CoA synthetase activity in gastrocnemius muscles of control and miDrp1^KO^ mice (*n* = 5 to 8) at 3 weeks post–*Dnm1l* deletion. (**I**) Triacylglycerol levels in gastrocnemius muscles of control (*n* = 8) and miDrp1^KO^ (*n* = 6) mice at 3 weeks post–*Dnm1l* deletion. Data are presented as means ± SEM; unpaired Student’s *t* test two tailed. *P < 0.05.

To determine the impact of timing of Drp1 deletion on muscle metabolism, we compared conventional and inducible Drp1-knockout models. In contrast to the reduced oxygen consumption rate (VO_2_) and energy expenditure (EE) observed in conventional mDrp1^HET^ mice, conditional miDrp1^HET^ mice exhibited no change in systemic metabolism, physical activity, or food and water intake in 4 weeks after *Dnm1l* gene deletion, relative to their respective control mice (Fig. 2A and fig. S2, A to G) (*19*). Furthermore, high-fat diet (HFD) feeding did not alter this wasting phenotype, suggesting that muscle wasting in miDrp1^KO^ mice is independent of caloric intake (fig. S2H). Although mDrp1^HET^ mice displayed impaired glucose and insulin tolerance, miDrp1^KO^ mice showed no alterations in plasma concentrations of insulin and leptin or glucose homeostasis; however, they demonstrated blunted counterregulatory responses to insulin, consistent with prior observations in partial Drp1 knockout models (Fig. 2, B to E) (*19*, *23*). Both mDrp1^HET^ and miDrp1^KO^ mice have significantly elevated plasma lactate, but unchanged succinate levels were observed only in miDrp1^KO^ mice. This may be explained by the reduced succinyl–coenzyme A (CoA) synthetase activity observed specifically in the skeletal muscle miDrp1^KO^ mice (Fig. 2, F to H) (*19*). Moreover, although mDrp1^HET^ mice show skeletal muscle lipid accumulation, no differences in muscle triglyceride or cholesterol ester content were detected between miDrp1^KO^ and control mice (Fig. 2I and fig. S2, I to L) (*19*).

### Progressive decline in mtDNA content following acute Drp1 deletion

**Fig. 3.**
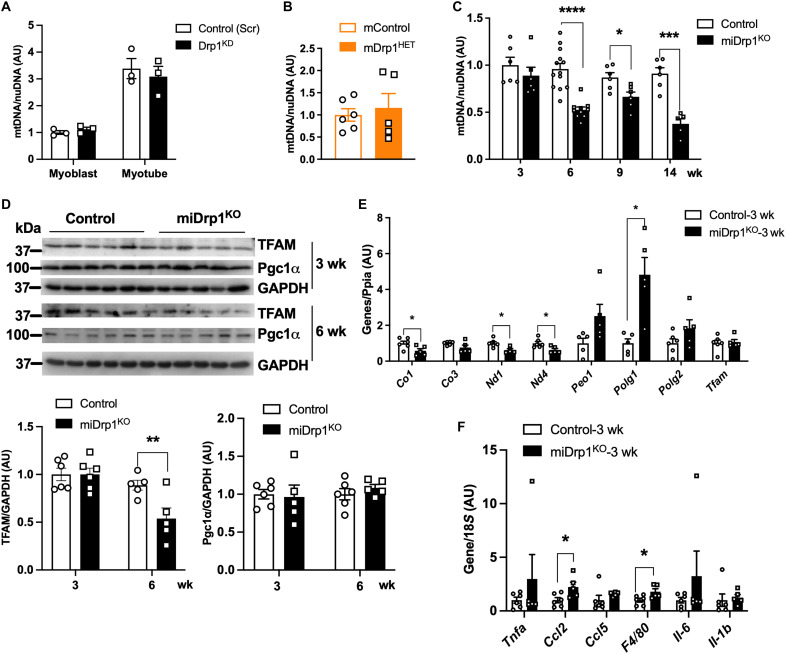
Acute Drp1 deletion drives progressive mtDNA depletion. (**A**) mtDNA content in control (Scr) and Drp1^KD^ C2C12 myoblasts and myotubes (*n* = 3). (**B**) mtDNA content in gastrocnemius muscles of 4- to 6-month-old male mControl (*n* = 6) and mDrp1^HET^ mice (*n* = 5). (**C**) mtDNA content in gastrocnemius muscles of control and miDrp1^KO^ mice at 3 weeks (control, *n* = 6; miDrp1^KO^, *n* = 6), 6 weeks (control, *n* = 13; miDrp1^KO^, *n* = 10), 9 weeks (control, *n* = 6; miDrp1^KO^, *n* = 6), and 14 weeks (control, *n* = 6; miDrp1^KO^, *n* = 5) post–*Dnm1l* deletion. (**D**) Western blot and the densitometric analysis (bottom) of TFAM and Pgc1α in quadriceps muscles of controls (*n* = 6) and miDrp1^KO^ mice (*n* = 5) at 3 and 6 weeks post–*Dnm1l* deletion. (**E**) mtDNA-encoded gene and mtDNA transcription and replication transcript in quadriceps muscles of controls (*n* = 6) and miDrp1^KO^ mice (*n* = 5) at 3 weeks post–*Dnm1l* deletion. (**F**) Inflammation gene transcript in quadriceps muscles of controls (*n* = 6) and miDrp1^KO^ mice (*n* = 5) at 3 weeks post–*Dnm1l* deletion. Data are presented as means ± SEM; unpaired Student’s *t* test two tailed. *P < 0.05; **P < 0.01; ***P < 0.001; ****P < 0.0001.

Maintaining balanced mitochondrial dynamics is essential for preserving mitochondrial genome integrity and preventing sterile inflammation triggered by mislocalized mtDNA and activation of intracellular DNA sensors (*13*). We therefore examined the impact of Drp1 deletion on mitochondrial genome integrity and associated stress responses. mtDNA content was unchanged in Drp1^KD^ C2C12 myoblast and myotubes and in the skeletal muscle of miDrp1^HET^ mice (Fig. 3, A and B). However, mtDNA content significantly decreased, starting at 6 weeks after *Dnm1l* deletion, in both female and male miDrp1^HET^ and miDrp1^KO^ mice (Fig. 3C and fig. S3, A and B). Mitochondrial transcription factor A (TFAM) protein levels were significantly decreased at 6 weeks after *Dnm1l* deletion, whereas peroxisome proliferator–activated receptor gamma coactivator 1-alpha (Pgc1α) protein levels remained unchanged (Fig. 3D). Alongside the decrease in mtDNA content at 6 weeks, mtDNA-encoded genes, including cytochrome c oxidase I (*Co1*), NADH-ubiquinone oxidoreductase chain 1 (*Nd1*), and NADH-ubiquinone oxidoreductase chain 4 (*Nd4*), were significantly down-regulated as early as 3 weeks postdeletion, preceding the drop in mtDNA content (Fig. 3E). Similarly, the expression of mtDNA-encoded genes was significantly reduced in the gastrocnemius muscles of miDrp1^HET^ mice compared to control mice 6 weeks postdeletion (fig. S3C). Expression of mitochondrial polymerase gamma 1 (*Polg1*), which encodes the catalytic subunit of mtDNA polymerase gamma, was elevated by nearly sixfold in miDrp1^KO^ versus control, potentially reflecting a compensatory response to impaired mtDNA replication (Fig. 3E). Our previous study shows that mitochondrial complex II dysfunction is an early event that occurs following skeletal muscle Drp1 deletion (*19*). In support of this notion, inhibition of mitochondrial complex II activity in C2C12 myotubes using 3-nitropropionic acid (3-NP), an irreversible inhibitor of complex II, recapitulated the reduction in mtDNA content and mtDNA-encoded gene expression observed after *Dnm1l* deletion (fig. S3, D and E).

We next examined whether acute Drp1 deletion in the skeletal muscle triggers an early inflammatory response. Expression of inflammatory genes chemokine (C-C motif) ligand 2 (*Ccl2*) and adhesion G protein–coupled receptor E1 (*F4/80*) was significantly increased in miDrp1^KO^ skeletal muscle at 3 weeks post–*Dnm1l* deletion, whereas the phosphorylated nuclear factor of kappa light polypeptide gene enhancer in B cell inhibitor (IκB) Ser^32^ protein levels remained unchanged (Fig. 3F and fig. S3F). Notably, inflammatory cytokines and chemokines remained unchanged at 3 weeks but were significantly elevated by 11 weeks after *Dnm1l* deletion in miDrp1^KO^ mice compared with control mice (fig. S3, G and H). Similarly, 3-NP treatment heightened inflammatory gene expression in C2C12 myotubes (fig. S3I).

### Acute Drp1 deletion enhances mitochondrial degradation followed by ER stress responses in the skeletal muscle

**Fig. 4.**
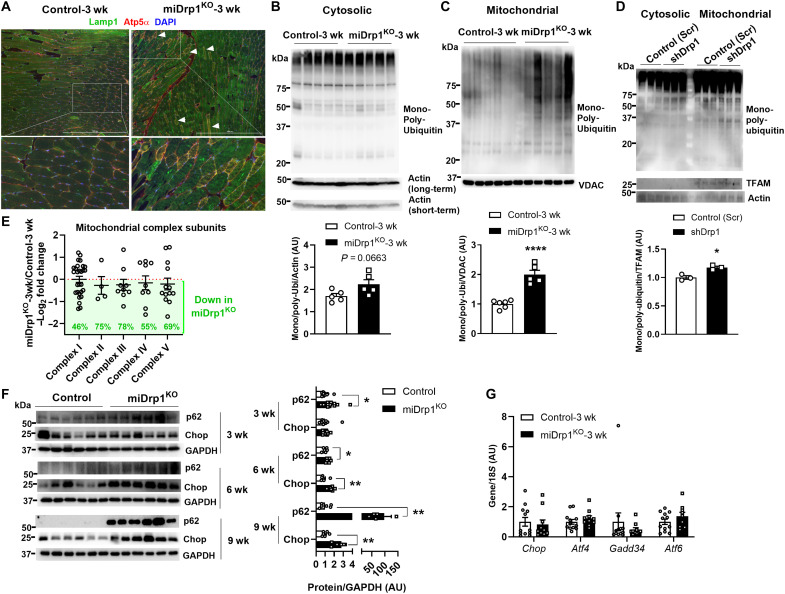
Drp1 deletion from the skeletal muscle promotes early activation of mitochondrial degradation. (**A**) Immunofluorescence analysis of the lysosomal marker Lamp1 and mitochondrial marker Atp5α in gastrocnemius muscles of male control and miDrp1^KO^ mice at 3 weeks post–*Dnm1l* deletion (scale bars, 1000 μm). Western blot and the densitometric analysis (bottom) of mono- and poly-ubiquitin protein abundance in the (**B**) cytosolic fraction (control, *n* = 6; miDrp1^KO^, *n* = 5) and (**C**) mitochondrial fraction (control, *n* = 5; miDrp1^KO^, *n* = 5) of gastrocnemius muscles at 3 weeks post–*Dnm1l* deletion. VDAC, voltage-dependent anion channel 1. (**D**) Western blot and the densitometric analysis (bottom) of mono- and poly-ubiquitin protein levels in the cytosolic fraction and mitochondrial fraction (*n* = 3) of C2C12 myotubes treated with lentiviral shRNA targeting *Dnm1l* for 96 hours to achieve Drp1 KD. (**E**) Mitochondrial complex–associated subunit abundance detected in isolated organelles from gastrocnemius muscles of miDrp1^KO^ (*n* = 6) versus control mice (*n* = 6) at 3 weeks post–*Dnm1l* deletion, as determined by MS. (**F**) Western blot and the densitometric analysis (right) of p62 and Chop in quadriceps muscles of controls and miDrp1^KO^ mice at 3 weeks (control, *n* = 6; miDrp1^KO^, *n* = 6), 6 weeks (control, *n* = 6; miDrp1^KO^, *n* = 6), and 9 weeks (control, *n* = 12; miDrp1^KO^, *n* = 11) post–*Dnm1l* deletion. (**G**) Gene expression of ER stress markers in quadriceps muscles of controls (*n* = 12) and miDrp1^KO^ (*n* = 10) mice at 3 weeks post–*Dnm1l* deletion. Data are presented as means ± SEM; unpaired Student’s *t* test two tailed. *P < 0.05; **P < 0.01; ****P < 0.0001.

The decline in mtDNA content prompted us to investigate whether Drp1 deletion alters mitophagy, a potential mechanism underlying mitochondrial depletion. Drp1 is essential for segregation of damaged mitochondria for mitophagy (*24*). Unexpectedly, we observed a notable overlap of lysosomal-associated membrane protein 1 (Lamp1, lysosomal marker) and ATP synthase F1 subunit α (Atp5α, mitochondrial marker) in the skeletal muscle of miDrp1^KO^ mice as early as 3 weeks post–*Dnm1l* deletion (Fig. 4A and fig. S4A), indicating increased lysosome-mitochondrion interactions. MitoQC is a fluorescence-based reporter that enables real-time assessment of mitophagy through pH-sensitive dual fluorescence signals (*25*). We observed an increased number of red-only fluorescence signals in the skeletal muscle of miDrp1^KO^ mice 3 weeks post–*Dnm1l* deletion versus control (fig. S4B), indicating enhanced mitochondrial degradation. To further validate the mitochondrial degradation in muscle of miDrp1^KO^ mice, we separated cytosolic and mitochondrial fractions of the skeletal muscle to assess mitochondrial ubiquitination. Consistent with increased lysosomal-mitochondrial colocalization, we detected increased abundance of mono-poly-ubiquitin–labeled proteins in the mitochondrial fraction. In contrast, the cytosolic fraction showed a modest upward trend that did not reach statistical significance (Fig. 4, B and C). Transient knockdown (KD) of Drp1 in C2C12 myotubes using lentiviral short hairpin RNA (shRNA) shows a consistent increase in mono-poly-ubiquitin protein levels in mitochondrial fractions (Fig. 4D and fig. S4C). Together, these findings suggest that acute Drp1 deletion elevates mitochondrial degradation in the skeletal muscle. To further support this, we next performed mass spectrometry (MS) profiling proteins in isolated mitochondria from the skeletal muscle of control and miDrp1^KO^ mice at 3 weeks post–*Dnm1l* deletion. MS confirmed lower mitochondrial Drp1 protein levels in miDrp1^KO^ muscles (fig. S4D). Mitochondrial proteomics enrichment analysis strongly linked impairment of mitochondrial oxidative metabolism with muscle dysfunction. Proteins associated with electron transport chain (ETC) complexes I, II, III, IV, and V were significantly reduced by 46, 75, 78, 55, and 69%, respectively (Fig. 4E).

Mitochondrial turnover by mitophagy relies heavily on macroautophagy (*26*). Sequestosome 1 (p62) is an autophagy adaptor protein that binds to ubiquitinated cargo and facilitates its degradation by interacting with microtubule-associated protein 1 light chain (LC3), thereby promoting autophagosome formation and lysosomal degradation (*27*, *28*). We examined p62 and LC3 protein abundance at 3, 6, and 9 weeks post–*Dnm1l* deletion. We observed a sustained elevation in p62 protein levels in the skeletal muscle of miDrp1^KO^ mice compared to control mice, beginning 3 weeks after *Dnm1l* deletion and persisting through 9 weeks (Fig. 4F). The relative level of LC3BII to LC3BI is used as a biomarker of autophagosome formation and autophagy flux (*29*). Elevated LC3BII protein levels were only observed at 9 weeks post–*Dnm1l* deletion (fig. S4, E to G), with similar findings for Pgc1α (fig. S4G). These findings indicate that acute Drp1 deletion rapidly triggers accumulation of p62 without concurrent changes in LC3 and mitochondrial biogenesis.

A previous study has shown that deletion of Drp1 induces endoplasmic reticulum (ER) stress at 70 days after *Dnm1l* gene deletion (*17*). We found that the gene expression of ER stress markers, including DNA damage-inducible transcript 3 (*Chop*), activating transcription factor 4 (*Atf4*), protein phosphatase 1 regulatory subunit 15A (*Gadd34*), and activating transcription factor 6 (*Atf6*), as well as protein levels of phosphorylated eukaryotic translation initiation factor 2 alpha (Eif2α), was unchanged in the skeletal muscle of miDrp1^KO^ mice at 3 weeks post–*Dnm1l* deletion (Fig. 4G and fig. S4H). C/EBP homologous protein (Chop) was elevated starting at 6 and 9 weeks post–*Dnm1l* deletion (Fig. 4F). These findings suggest that elevated ER stress is not an early response to Drp1 deletion.

Kelch-like ECH-associated protein 1 (Keap1), a substrate adaptor of the Cullin 3–RING E3 ubiquitin ligase complex, is recruited to mitochondria by p62 and facilitates Parkin-independent mitophagy in the liver (*30*). However, Keap1 protein levels remained unchanged in the mitochondrial fraction of skeletal muscle between control and miDrp1^KO^ mice at 3 weeks post–*Dnm1l* deletion (fig. S4I), suggesting that Keap1 may not play a major role in early mitophagic responses to Drp1 deletion in the skeletal muscle.

### Acute Drp1 deletion activates Parkin-mediated mitochondrial degradation

**Fig. 5.**
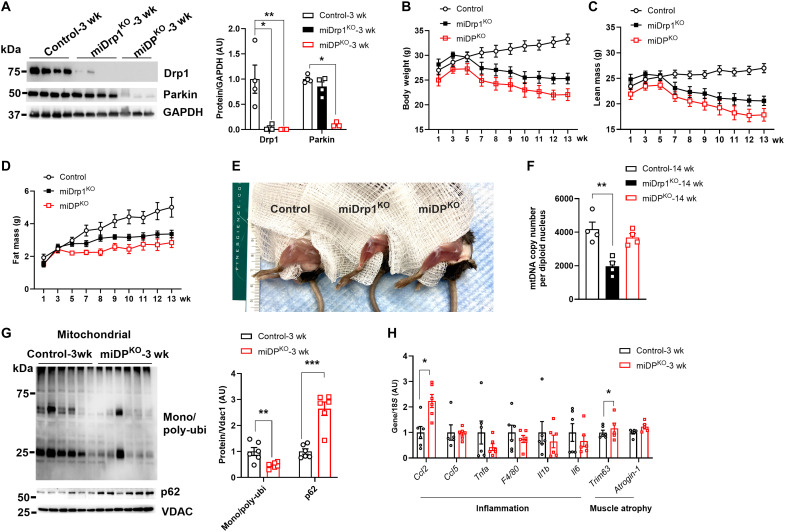
Parkin is required for mitochondrial degradation but not for muscle wasting in Drp1-deficient skeletal muscle. (**A**) Western blot and the densitometric analysis (right) of Drp1 and Parkin in quadriceps muscles of controls (*n* = 4), miDrp1^KO^ (*n* = 4), and miDP^KO^ (*n* = 3) mice. (**B**) Body weight, (**C**) lean mass, and (**D**) fat mass of controls (*n* = 4), miDrp1^KO^ (*n* = 4), and miDP^KO^ (*n* = 3) mice post–TM injection. (**E**) Hindlimbs of male controls, miDrp1^KO^, and miDP^KO^ mice at 14 weeks post–*Dnm1l* deletion. (**F**) mtDNA content in gastrocnemius muscles of controls (*n* = 4), miDrp1^KO^ (*n* = 4), and miDP^KO^ (*n* = 4) mice at 14 weeks post–*Dnm1l* deletion examined by digital PCR. (**G**) Western blot and the densitometric analysis (right) of mono- and poly-ubiquitin protein levels in mitochondrial fractions of quadriceps muscles from control (*n* = 6) and miDrp1^KO^ (*n* = 6) mice at 3 weeks post–*Dnm1l* deletion. (**H**) Inflammation and muscle atrophy gene expression in quadriceps muscles of controls (*n* = 6) and miDP^KO^ (*n* = 6) mice at 3 weeks post–*Dnm1l* deletion. Data are presented as means ± SEM; unpaired Student’s *t* test two tailed. *P < 0.05; **P < 0.01; ***P < 0.001.

Parkin (encoded by the *Park2* gene), an E3 ubiquitin ligase, plays an essential role in mitophagy by ubiquitinating outer mitochondrial membrane proteins on damaged mitochondria (*31*). However, mitophagy can occur through both Parkin-dependent and Parkin-independent pathways (*32*–*34*). In the skeletal muscle, we observed that Parkin protein levels were reduced in mDrp1^HET^ mice but not miDrp1^KO^ mice (fig. S5, A and B). These findings suggest that mitochondrial degradation in miDrp1^KO^ muscle could be mediated by Parkin-dependent mechanisms. To validate this, we generated a TM-inducible Drp1 and Parkin double-knockout mouse model (miDP^KO^). As expected, Drp1 protein levels were markedly reduced in the skeletal muscle of both miDrp1^KO^ and miDP^KO^ mice compared to control mice, whereas Parkin protein levels were only decreased in miDP^KO^ mice (Fig. 5A). miDP^KO^ still exhibited a muscle wasting phenotype similar to miDrp1^KO^ mice, with significant reductions in both quadricep and gastrocnemius muscle mass (Fig. 5, B to E, and fig. S5C). Notably, both digital polymerase chain reaction (PCR) and real-time PCR [quantitative (qPCR)] analyses revealed that Parkin deletion preserved mtDNA content in the skeletal muscle of miDP^KO^ mice compared with control mice (Fig. 5F and fig. S5D). In addition, mono- and poly-ubiquitinated protein abundance was significantly decreased in isolated mitochondrial fractions from miDP^KO^ skeletal muscle (Fig. 5G). In contrast, elevated p62 protein levels were observed in both isolated mitochondrial fractions and total muscle lysates as early as 3 weeks post–*Dnm1l* and *Park2* deletion (Fig. 5G and fig. S5E). These findings indicate that Parkin is a key mediator of mitochondrial degradation in miDrp1^KO^ mice. Consistent with the muscle atrophy phenotype, the gene expression of pro-inflammatory cytokine *Ccl2* and atrophy marker muscle RING finger protein 1 (*Trim63*) was increased in the skeletal muscle of miDP^KO^ versus control (Fig. 5H).

To better clarify Parkin’s role in the skeletal muscle, we generated a TM-inducible Parkin knockout mouse model (miParkin^KO^). Body weight of miParkin^KO^ mice remained unchanged under normal chow (NC) feeding but was significantly increased following an HFD feeding versus control (fig. S5, F and G), indicating that Parkin deletion alone is insufficient to drive muscle atrophy.

### Drp1 deletion reduces Nur77 expression to promote muscle atrophy

**Fig. 6.**
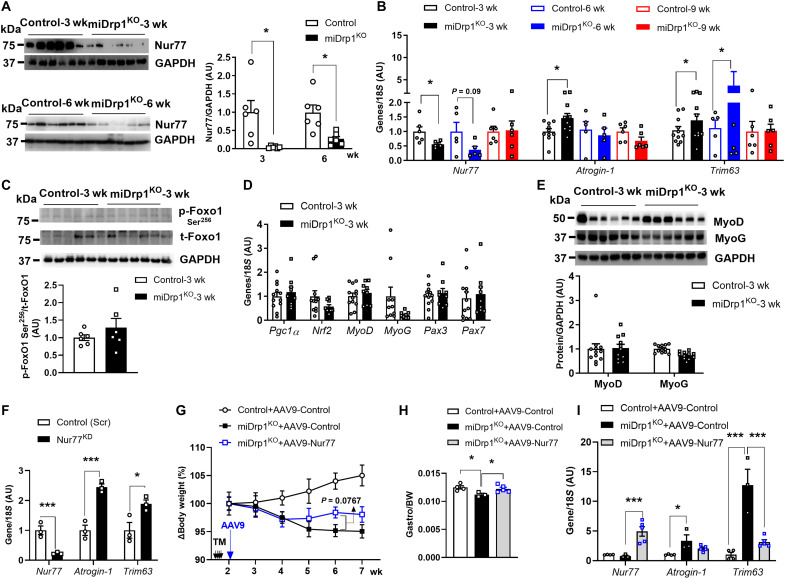
Acute Drp1 deletion suppresses Nur77 expression and promotes muscle atrophy. (**A**) Western blot and the densitometric analysis (right) of Nur77 in quadriceps muscles of controls (*n* = 6) and miDrp1^KO^ (*n* = 6) mice at 3 and 6 weeks post–*Dnm1l* deletion. (**B**) *Nur77*, *Atrogin-1*, and *Trim63* gene expression in quadriceps muscles of controls and miDrp1^KO^ mice at 3, 6, and 9 weeks post–*Dnm1l* deletion (*n* = 5 to 11). (**C**) Western blot and the densitometric analysis (bottom) of phospho-FoxO1 Ser^256^ and FoxO1 in quadriceps muscles of controls (*n* = 6) and miDrp1^KO^ (*n* = 6) mice at 3 weeks post–*Dnm1l* deletion. (**D**) Myogenesis gene expression in quadriceps muscles of controls (*n* = 12) and miDrp1^KO^ (*n* = 9) mice at 3 weeks post–*Dnm1l* deletion. (**E**) Western blot and the densitometric analysis (bottom) of MyoD and MyoG in quadriceps muscles of controls (*n* = 12) and miDrp1^KO^ (*n* = 11) mice at 3 weeks post–*Dnm1l* deletion. (**F**) *Nur77*, *Atrogin-1*, and *Trim63* gene expression in control (Scr) and Nur77^KD^ C2C12 myotubes (*n* = 3). (**G**) Delta body weight, (**H**) gastrocnemius muscle–to–body weight ratio, and (**I**) *Nur77*, *Atrogin-1*, and *Trim63* gene expression in gastrocnemius muscles of control mice injected with AAV9-HSA-control (*n* = 4), miDrp1^KO^ mice injected with AAV9- HSA-control (*n* = 3), and miDrp1^KO^ mice injected with AAV9- HSA-Nur77 (*n* = 5) at the indicated time. Data are presented as means ± SEM; unpaired Student’s *t* test two tailed. *P < 0.05; ***P < 0.001.

Among the early transcriptional changes identified by RNA-seq analysis of muscle from mice 3 weeks after *Dnm1l* deletion, a significant reduction in the expression of *Nur77* was observed. Nur77 deficiency reduces muscle mass and myofiber size, primarily due to the loss of inhibitory regulation on muscle atrophy markers muscle atrophy F-box (*Atrogin-1*) and *Trim63* gene expression (*21*, *22*). Consistent with RNA-seq data, both Nur77 transcript and protein levels were significantly reduced in miDrp1^KO^ skeletal muscle as early as 3 weeks post–*Dnm1l* deletion, with the reduction in protein levels persisting up to 6 weeks (Fig. 6A). Reduction of *Nur77* gene expression was also observed in miDP^KO^ mice at 3 weeks post–*Dnm1l* deletion but not in mDrp1^HET^ mice (fig. S6A). Moreover, we found that 3-NP (complex II inhibitor) treatment also significantly reduced *Nur77* gene expression in C2C12 myotubes (fig. S6B). Notably, the gene expression of *Atrogin-1* and *Trim63* was also up-regulated in miDrp1^KO^ mice at both 3 and 6 weeks post–*Dnm1l* deletion versus control (Fig. 6B). FoxO1 and FoxO3a are key transcription factors that promote muscle atrophy by activating the expression of *Atrogin-1* and *Trim63* (*35*). However, neither the phosphorylation of FoxO1 nor FoxO3a were changed in miDrp1^KO^ mice compared to control mice (Fig. 6C and fig. S6C).

Muscle atrophy can trigger compensatory myogenesis. However, we observed no difference in the gene expression of key myogenesis regulators, including paired box 3 (*Pax3*), paired box 7 (*Pax7*), myoblast determination protein 1 (*MyoD*), and myogenin (*MyoG*), between the genotypes at both 3 and 6 weeks following *Dnm1l* deletion (Fig. 6D and fig. S6D). Consistently, both MyoD and MyoG protein levels were unchanged in the skeletal muscle of miDrp1^KO^ mice versus control at 3 weeks (Fig. 6E and fig. S6E), indicating that muscle atrophy in this mouse model occurs independently of compensatory myogenic activation during this early phase following *Dnm1l* deletion.

To further determine whether Nur77 deletion induces muscle atrophy signaling in muscle cells, we performed transient knockdown (KD) of Nur77 in differentiated C2C12 mouse myotubes using shRNA. Knockdown of *Nur77* increased both *Atrogin-1* and *Tirm63* expression compared with control scramble (Scr) cells (Fig. 6F and fig. S6F). However, *Nur77* KD did not alter mitophagy signal and mtDNA content in cells (fig. S6, G and H). *Nur77* KD elevated p62 protein levels in the whole-cell lysates and showed a similar trend in the mitochondrial fractions (fig. S6, I and J). To confirm the protective role of Nur77 in safeguarding against muscle atrophy in miDrp1^KO^ mice, we overexpressed Nur77 in the skeletal muscle by intramuscular injection of AAV9-HSA-Nur77 into both gastrocnemius muscles at 2 weeks after *Dnm1l* deletion. Nur77 overexpression increased the overall body weight and the gastrocnemius muscle–to–body weight ratio while markedly suppressing *Trim63* expression (Fig. 6, G to I). These data position Nur77 as a critical transcriptional target downstream of Drp1 signaling, with a key role in maintaining muscle mass.

### Acute Drp1 deletion rapidly inhibits Erk1/2 signaling and downstream Nur77 action

**Fig. 7.**
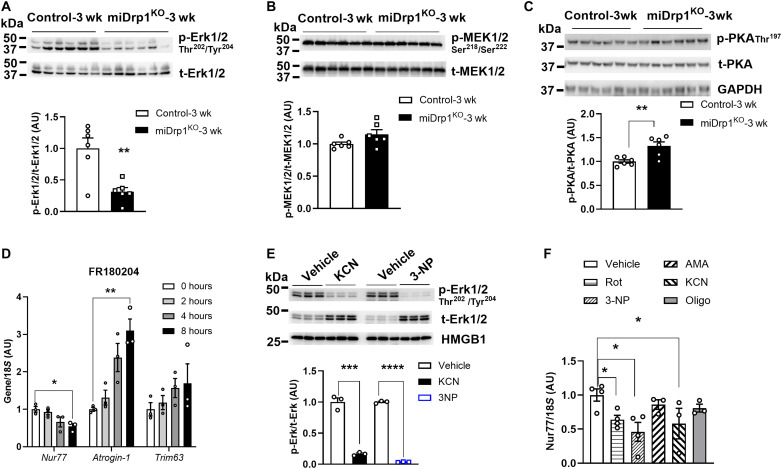
Acute Drp1 deletion disrupts mitochondrial function and Erk1/2-Nur77 signaling in the skeletal muscle. (**A**) Western blot and densitometric analysis (bottom) of protein levels for phosphorylated Erk1/2 Thr^202^/Tyr^204^, (**B**) phosphorylated MEK1/2 Ser^218^/Ser^222^, and (**C**) phosphorylated PKA Thr^197^ in quadriceps muscles of controls (*n* = 6) and miDrp1^KO^ (*n* = 6) mice at 3 weeks post–*Dnm1l* deletion. (**D**) Inhibition of Erk1/2 activity by 20 μM FR180204 decreased *Nur77* gene expression but elevated *Atrogin-1* and *Trim63* gene expression in C2C12 myotubes (*n* = 3). (**E**) Western blot of phosphorylated Erk1/2 Thr^202^/Tyr^204^ and Erk1/2 protein levels in C2C12 myotubes treated with KCN (complex IV inhibitor, 16 hours) and 3-NP (complex II inhibitor, 16 hours). (**F**) *Nur77* gene expression in C2C12 myotubes treated with indicated mitochondrial complex inhibitors. Data are presented as means ± SEM; unpaired Student’s *t* test two tailed. *P < 0.05; **P < 0.01; *** *P* < 0.001; **** *P* < 0.0001.

Molecular function enrichment analysis of RNA-seq data from the skeletal muscle of miDrp1^KO^ mice showed down-regulation of cyclic adenosine monophosphate (cAMP) binding and protein kinase A (PKA) binding 3 weeks following *Dnm1l* deletion (fig. S7A). Erk1/2, a key downstream target of cAMP-PKA signaling, is known to phosphorylate Drp1 at Ser^616^, promoting mitochondrial fission (*36*, *37*). Erk1/2 phosphorylation was markedly reduced in muscle from miDrp1^KO^ mice at 3 weeks after *Dnm1l* deletion, despite identical phosphorylation levels of its upstream regulator, mitogen-activated protein kinase kinase 1/2 (MEK1/2) (Fig. 7, A and B). Notably, PKA phosphorylation and PKA-phosphorylated substrates were significantly increased in the skeletal muscle of miDrp1^KO^ mice at 3 weeks after Drp1 deletion (Fig. 7C and fig. S7B). Given the established role of Erk1/2 in transcriptional regulation of genes involved in muscle remodeling, we next examined the impact of Erk1/2 signaling on *Nur77* expression. We treated C2C12 myotubes with FR180204, a selective Erk1/2 inhibitor, which significantly reduced *Nur77* expression while increasing *Atrogin-1* and *Tirm63* levels in C2C12 myotubes (Fig. 7D). These findings link Erk1/2 to Nur77-mediated transcriptional control in the context of Drp1 deletion in the skeletal muscle.

To investigate whether mitochondrial complex dysfunction suppresses Erk1/2-Nur77 signaling, we treated C2C12 myotubes with inhibitors targeting each respiratory complex: rotenone (Rot; complex I), 3-NP (complex II), antimycin A (AMA; complex III), potassium cyanide (KCN; complex IV), and oligomycin (Oligo; complex V). All inhibitors effectively decreased Erk1/2 phosphorylation in C2C12 myotubes, although not all complex inhibitors reduced Nur77 gene expression (Fig. 7, E and F, and fig. S7, C and D), suggesting that mitochondrial dysfunction contributes to Erk1/2-Nur77 suppression. Collectively, these findings demonstrate that Erk1/2-Nur77 acts as a central node linking mitochondrial dysfunction to muscle atrophy.

### Clenbuterol mitigates muscle atrophy in miDrp1^KO^ mice

**Fig. 8.**
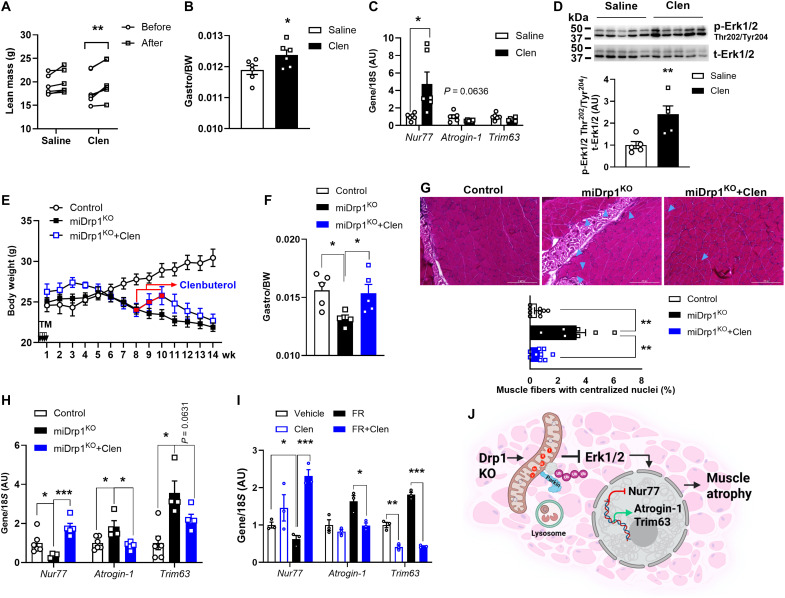
Clenbuterol mitigates muscle atrophy in miDrp1^KO^ mice. One week of Clen (30 mg/liter) treatment in drinking water increased. (**A**) Lean mass, (**B**) relative gastrocnemius muscle weight, (**C**) *Nur77* gene expression, and (**D**) Erk1/2 Thr^202^/Tyr^204^ phosphorylation in gastrocnemius muscles of WT mice (*n* = 5 to 6). (**E**) Body weight of control (*n* = 6), miDrp1^KO^ (*n* = 8), and miDrp1^KO^+Clen (30 mg/liter; *n* = 4) after TM injection and Clen treatment. In a second cohort, at 8 weeks post–TM injection, control and miDrp1^KO^ mice consume regular water, whereas miDrp1^KO^+Clen mice received Clen in the drinking water for 1 week. One week of Clen treatment (**F**) restored relative gastrocnemius muscle weight (*n* = 4 to 5), (**G**) reduced centrally nucleated muscle fibers in gastrocnemius muscles (blue arrows indicate fibers with centralized nuclei; scale bars, 200 μm; *n* = 3), and (**H**) increased *Nur77* gene expression and suppressed *Atrogin-1* and *Tirm63* gene expression (*n* = 4 to 6). (**I**) *Nur77*, *Atrogin-1*, and *Trim63* gene expression in FR180204 (10 μM, 24 hours) pretreated C2C12 myotubes with and without Clen (30 ng/ml) for 1 hour (*n* = 3). (**J**) Schematic overview of acute Drp1 deletion promotes Parkin-mediated mitochondrial degradation and controls skeletal muscle mass through the Erk1/2-Nur77 pathway. Data are presented as means ± SEM; unpaired Student’s *t* test two tailed. *P < 0.05; **P < 0.01; ***P < 0.001.

Clenbuterol (Clen), a β_2_-adrenergic receptor agonist, increases muscle mass by activating the cAMP/PKA pathway, which, in turn, stimulates Erk1/2 signaling, promoting protein synthesis and reducing muscle degradation (*38*–*41*). Treatment of wild-type (WT) mice with Clen in drinking water for 1 week significantly increased lean mass, particularly in the gastrocnemius muscle, up-regulated *Nur77* gene expression and protein level (*P* = 0.0871), and showed a trend toward reduced *Atrogin-1* expression, accompanied by a marked increase in Erk1/2 phosphorylation in gastrocnemius muscles (Fig. 8, A to D, and fig. S8A). To determine whether Clen could mitigate muscle wasting in miDrp1^KO^ mice, we administered it in drinking water for 2 weeks beginning 8 weeks post–*Dnm1l* deletion. Clen-treated miDrp1^KO^ mice gained an average of ~1.7 g of body weight versus miDrp1^KO^ mice receiving regular drinking water (Fig. 8E). Notably, even after 1 week of treatment, Clen markedly increased the relative gastrocnemius muscle weight, reduced the proportion of fibers with centralized nuclei, enhanced Erk1/2 phosphorylation, and decreased the expression of muscle atrophy–related genes *Atrogin-1* and *Trim63*, compared with miDrp1^KO^ mice receiving regular drinking water (Fig. 8, F to H, and fig. S8, B and C). To further confirm the involvement of Erk1/2 signaling, we examined C2C12 myotubes, where Clen counteracted the effects of the Erk1/2 inhibitor FR180204, restoring *Nur77* expression while preventing the induction of *Atrogin-1* and *Trim63* (Fig. 8I). Together, these findings demonstrate that Clen restores Erk1/2-Nur77 signaling to counteract muscle atrophy in miDrp1^KO^ mice.

## DISCUSSION

Mitochondrial myopathies are a genetically heterogeneous class of disorders characterized by impaired oxidative metabolism, progressive muscle weakness, and atrophy (*42*–*44*). Given the critical role of mitochondria in muscle health, mitochondrial quality control is essential for maintaining skeletal muscle function and mass, with Drp1 playing a key role in regulating mitochondrial fission and turnover (*45*, *46*). Our previous study of mice with conventional heterozygous muscle-specific *Dnm1l* knockout (mDrp1^HET^) mice shows that Drp1 promotes Sdhaf2-mediated assembly of mitochondrial complex II, thereby enhancing lipid oxidation and insulin action (*19*). In contrast to the conventional Drp1 deletion model, TM-inducible skeletal muscle-specific *Dnm1l* knockout (miDrp1^KO^) mice develop severe muscle wasting and weakness, primarily through activation of the unfolded protein response and disruption of Ca^2+^ homeostasis (*17*). In the current study, we examined both models simultaneously and found that, despite their distinct phenotypes, both models exhibited elevated plasma lactate and reduced complex II activity, underscoring a shared defect in mitochondrial respiration compared with their controls. Succinate levels were elevated in the skeletal muscle of mDrp1^HET^ mice but remained unchanged in miDrp1^KO^ mice, as a likely consequence of diminished upstream succinyl-CoA synthetase activity in miDrp1^KO^ mice compared to controls. These findings highlight the central role of Drp1 in preserving complex II function and demonstrate that the mechanisms disrupted depend on the timing of Drp1 deletion in each model. Although a previous study suggests that ER stress contributes to muscle atrophy following Drp1 deletion (*17*), our findings indicate that ER stress is not an early response to Drp1 deletion but rather a secondary consequence.

mtDNA content, which declines with aging and correlates positively with lean mass in humans (*47*–*49*), is influenced by mitochondrial transcription factors such as TFAM and Pgc1α (*50*, *51*). We observed a rapid decline in mtDNA-encoded gene expression together with reduced mtDNA content in miDrp1^KO^ muscle following *Dnm1l* deletion. Although TFAM and Pgc1α transcript levels were unchanged, TFAM protein abundance was diminished and Pgc1α protein levels were not elevated. These findings suggest that the loss of mtDNA in Drp1-deficient muscle is driven primarily by enhanced mitochondrial degradation rather than impaired mitochondrial biogenesis. Elevated Pgc1α protein levels were observed after mtDNA content reduction likely reflecting a compensatory response. These findings underscore the critical role of Drp1 in maintaining mtDNA stability. Beyond the canonical mitophagy pathway, Parkin regulates the biogenesis of mitochondrial-derived vesicles (MDVs) but also governs their trafficking to the endolysosomal compartment for degradation (*52*–*55*). In Drp1-deficient muscle, elevated p62 without alterations of LC3 protein abundance is consistent with impairment of canonical mitophagy. However, enhanced colocalization of mitochondria with lysosomes and reduced mtDNA content implicates that mitochondrial degradation occurs through a compensatory pathway that circumvents the blockade of canonical mitophagy. The reversal of mitochondrial ubiquitination and mtDNA depletion in the Drp1 and Parkin double-knockout model indicates the primacy of Parkin in compensatory mitochondrial degradation. Although mtDNA content was restored in miDP^KO^ mice, a severe muscle atrophy phenotype was still observed, indicating that mtDNA depletion is a parallel consequence rather than the primary driver of muscle atrophy. Consistent with findings in liver-specific Drp1 knockout mice (*30*, *56*), p62 total protein and mitochondrial ubiquitination were increased in skeletal muscle–specific Drp1 knockout mice. However, in contrast to the liver, where Drp1 deletion promotes Parkin-independent mitophagy, acute loss of Drp1 from the skeletal muscle triggers a Parkin-dependent mitochondrial degradation, highlighting a tissue-specific role of Drp1 in regulating mitochondrial quality control.

Nur77 is a transcriptional regulator of muscle mass, in part by suppressing the expression of muscle atrophy–related genes such as *Atrogin-1* and *Trim63* (*21*, *22*, *57*). We identified Nur77 as an early response gene to Drp1 deletion in the skeletal muscle, with its expression inversely correlating with *Atrogin-1* and *Trim63* and positively correlating with muscle mass. Knockdown of Nur77 increased *Atrogin-1* and *Trim63* expression without affecting mitophagy or mtDNA content. These divergent outcomes in mice versus cells may reflect the shorter duration of knockdown in vitro, which may have been insufficient to affect mitophagy.

We previously showed that Drp1 deletion specifically impairs complex II activity in both conventional and inducible Drp1 knockout mouse models (*19*). Inhibition of mitochondrial complex II by 3-NP recapitulated the decline in mtDNA content and mtDNA-encoded gene expression and suppressed *Nur77* expression, suggesting that complex II dysfunction contributes, at least partially, to the mitochondrial defects observed in the skeletal muscle of Drp1-null mice.

In the skeletal muscle, Erk1/2 integrates mechanical and metabolic cues to regulate growth and regeneration and phosphorylates Drp1 at Ser^616^ to promote mitochondrial fission (*58*–*62*). Acute Drp1 deletion markedly reduced Erk1/2 phosphorylation, suggesting that mitochondrial dysfunction caused by impaired fission may retrogradely suppress Erk1/2 signaling. Supporting this idea, inhibition of mitochondrial complexes I, II, and IV, but not III or V, suppressed Erk1/2 phosphorylation and selectively reduced Nur77. Because Erk1/2 is a known regulator of *Nur77* transcription (*63*, *64*), these findings indicate that ETC dysfunction converges on the Erk1/2-Nur77 axis to regulate muscle atrophy pathways. In line with prior studies showing that Clen (β_2_-adrenergic receptor agonist) rapidly increases Erk phosphorylation and *Nur77* expression (*65*, *66*), Clen treatment restored muscle weight in miDrp1^KO^ mice, suggesting that activation of Erk1/2 signaling may partially compensate for the loss of Drp1 and mitigate muscle atrophy. Together, these findings support targeting the Erk1/2-Nur77 pathway to preserve muscle mass in the context of mitochondrial dysfunction.

Both clinical and physiological contexts highlight the urgency of understanding mechanisms that preserve muscle mass. Potent glucagon-like peptide-1 (GLP-1) receptor agonists, which highly effective for weight reduction, can cause substantial loss of lean muscle mass (*67*–*69*). Similarly, aging-associated declines in Erk1/2 signaling in the skeletal muscle are linked to sarcopenia, the progressive loss of muscle mass and strength (*70*). These observations underscore the need to define the molecular mechanisms that promote muscle resilience. Our study identifies the Erk1/2-Nur77 signaling axis as an early target of disruption following acute Drp1 deletion and establishes a mechanistic link between mitochondrial dysfunction and transcriptional activation of muscle atrophy genes (Fig. 8J). Together, these findings define a unifying mitochondrial mechanism through which Drp1 insufficiency compromises muscle mass and reveal avenues for therapeutic intervention aimed at preserving muscle mass and enhancing muscle function.

## MATERIALS AND METHODS

### Sex as a biological variable

We examined both male and female mice and observed comparable muscle atrophy in both sexes; thus, subsequent experiments were performed using male mice.

### Animal

All animal care and experimentation procedures were approved by the University of California, Los Angeles, Institutional Animal Care and Use Committee (ARC-2020-187). Mice were maintained on a 12-hour light/12-hour dark cycle from 6:00 a.m. to 6:00 p.m. at ambient temperature (~22°C) with controlled humidity (~45%) in pathogen-free conditions. Food consumption, mouse activity, and health were monitored daily by the Division of Laboratory Animal Medicine at UCLA. Mice were euthanized by isoflurane overdose followed by cervical dislocation before organ harvest. This is an approved method according to the recommendations of the panel on Euthanasia of the American Veterinary Medical Association. The floxed Drp1 mice and floxed Parkin mice (gifts from H. Sesaki and T. M. Dawson, respectively, Johns Hopkins University) were first backcrossed to C57BL/6J mice for at least 10 generations. Then, the floxed Drp1 mice were crossed with a muscle-specific Cre transgenic mouse line (MCK-Cre, the Jackson Laboratory, no. 006475) to generate muscle-specific heterozygous Drp1-knockdown mice mDrp1^HET^ or with a TM-inducible, human *ACTA1* promoter driven Cre mouse line (HSA-MerCreMer, the Jackson Laboratory, no. 025750) to generate inducible skeletal muscle Drp1 knockout mice (miDrp1^KO^). TM-inducible skeletal muscle–specific Drp1 and Parkin knockout mice (miDP^KO^) were generated by crossing miDrp1^KO^ mice with Parkin flox mice. Using the same strategy, we also generated TM-inducible skeletal muscle–specific Parkin knockout mice (miParkin^KO^). Control (flox/+ or flox/flox), miDrp1^KO^, miDP^KO^, and miParkin^KO^ mice at 3 months old were intraperitoneally injected with TM (T5648, Sigma-Aldrich) (50 mg/kg per day) for 4 days. Control (flox/+ or flox/flox), mDrp1^HET^, miDrp1^KO^, and miParkin^KO^ male mice were maintained on an NC diet (LabDiet, 5053) or 45% HFD (Research Diets, D12451) and studied after 6 hours fasting. Body mass was examined by nuclear magnetic resonance (NMR) scanning. All mice studied were about 3 to 6 months of age. Blood from 6-hour-fasted mice was analyzed for circulating factors: glucose (HemoCue), inflammatory cytokines, and chemokines (Meso Scale Discovery, CA, catalog no. K15048D). Intraperitoneal insulin tolerance tests [ITTs; insulin (Humulin R), 0.7 U/kg] and intraperitoneal glucose tolerance tests (GTTs; dextrose, 1 g/kg) were performed on the same cohort of mice after 1 week of recovery between tests. Grip strength of mice was measured using a grip strength meter (Harvard Apparatus). Briefly, mouse was gently placed on a stable surface with forelimbs grasping the grid bar of the meter. Once a firm grip was established, the animal was carefully pulled backward by the tail with consistent force until it released the bar (*20*). The values on the meter were recorded. Clen (Sigma-Aldrich, C5423) was mixed in the drinking water (30 mg/liter) and provided at the indicated time points (*71*, *72*).

### Metabolic analysis

Body fat and lean mass were determined by NMR (Bruker). Oxygen consumption, carbon dioxide production, food and water consumption, and ambulatory movement were determined in NC-fed male mice using metabolic chambers (Oxymax metabolic chambers, Columbus Instruments).

### AAV injection

AAV9-HSA-mitoQC [1.2 × 10^11^ genome copies (gc) per mouse, Vector Biolabs, 221219#63] dissolved in 50 μl of saline was injected into both control and miDrp1^KO^ mice via intravenous injection 3 weeks after *Dnm1l* deletion. Subsequent studies were conducted on these mice 4 weeks later. AAV9-HSA-Nur77 (5 × 10^11^ gc per mouse, VectorBuilder, AAV9MP, VB241104-1505thm) dissolved in 50 μl of saline was injected into both sides of the gastrocnemius muscle of control and miDrp1^KO^ mice via intramuscular injection 2 weeks after *Dnm1l* deletion.

### Hematoxylin and eosin staining

Hematoxylin and eosin (H&E) staining was processed as described previously (*73*). Briefly, muscles were embedded in the Tissue-Plus O.C.T. compound (Fisher Scientific, #23-730-571) and then flash-frozen in liquid nitrogen–cooled isopentane. Transverse cryosections (10 μm) were collected on positively charged glass slides (Thermo Fisher Scientific) and stored at −80°C until use. For staining, sections were equilibrated to room temperature for 15 min, incubated in hematoxylin for 5 min, rinsed in distilled water for 2 min, and counterstained with eosin for 5 min. Slides were then sequentially dehydrated in 70, 80, 90, and 100% ethanol, cleared in xylene for 10 min, and mounted with Permount mounting medium. Muscle fiber CSA was quantified using the ImageJ software (NIH).

### Fluorescence immunohistochemistry

Formalin-fixed skeletal muscles were sectioned and stained for fluorescence immunohistochemistry. Following deparaffinization, rehydration, heat-induced epitope retrieval, and permeabilization, slides were blocked with 5% bovine serum albumin (BSA) and incubated with the Lamp1 antibody overnight followed by the goat anti-rabbit IgG Alexa Fluor 488 overnight inside a humidified chamber at 4°C. After BSA blocking, the slides were incubated with Atp5α antibody overnight and followed by the goat anti-mouse IgG Alexa Fluor 568 overnight at 4°C again. After washing in phosphate-buffered saline (PBS), coverslips were mounted in mounting medium (H-1000, Vector Labs) on a glass slide. Images were captured under a BioTek Lionheart LX automated microscope (Agilent).

### mtDNA isolation and quantification

Frozen gastrocnemius muscles were powdered in liquid nitrogen using a mortar and pestle. Total DNA was extracted by proteinase K digestion, phenol/chloroform extraction, and ammonium acetate precipitation. Total DNA quantity and quality were measured using spectrophotometry at A230, A260, and A280 (Thermo Fisher Scientific NanoDrop One^C^). Primers for mtDNA-encoded cytochrome c oxidase III (mtCO3) for mtDNA and 18*S* ribosomal RNA (18*S*) for nuclear DNA (nDNA) were used to assess mtDNA content. The ratio of mtCO3 to 18*S* was used to determine mtDNA content (*74*). For digital PCR, a 5′ nuclease cleavage assay specific for the mouse mitochondrial minor arc ND1 gene was used to quantitate total WT mtDNA copy number using a chip-based dPCR approach. Samples were diluted to the manufacturer’s recommended target range (200 to 2000 copies/μl). Digital PCR was used to quantify copy number for nDNA and mtDNA using as described before (*75*). Digital PCR cycling conditions included Taq polymerase activation at 95°C for 10 min, 40 cycles of denaturation at 94°C for 30 s, and annealing/extension at 60°C for 2 min. mtDNA copy numbers per microliter and the threshold were determined using the QuantStudio 3D Analysis Suite Cloud Software (version 3, Thermo Fisher Scientific).

### Mitochondria isolation

Mitochondria were isolated from gastrocnemius muscles using a Dounce homogenizer and Mitochondria Isolation Kit for Tissue (Thermo Fisher Scientific, catalog no. 89801) (*76*). Briefly, gastrocnemius muscles were washed in ice-cold PBS, Dounce homogenized, and centrifuged at 700*g* for 10 min at 4°C. The supernatant was centrifuged at 12,000*g* for 15 min at 4°C. The pellet was washed and centrifuged at 12,000*g* for 5 min at 4°C. Isolated mitochondria were mixed with 1x radioimmunoprecipitation assay (RIPA) lysis buffer (Millipore, 20-188) with protease inhibitor (cOmplete EDTA-free, Roche) and then subjected to bath sonication for 1 min (Bioruptor Pico, Diagenode Inc.). Approximately 20 to 40 μg of the protein from mitochondrial lysates, collected after centrifugation at 12,000*g* for 15 min at 4°C, was heated in Laemmli sample buffer (Bio-Rad) containing 2-mercaptoethanol (Sigma-Aldrich, #M3148) at 95°C for 5 min before immunoblot analysis.

### Cell culture and treatments

C2C12 cells (American Type Culture Collection, CRL-1772, RRID: CVCL_0188) were maintained in high-glucose Dulbecco’s modified Eagle’s medium (DMEM) supplemented with 10% fetal bovine serum and penicillin/streptomycin. To obtain C2C12 myotubes, cells were allowed to reach confluence, and the medium was switched for high-glucose DMEM supplemented with 2% horse serum and penicillin/streptomycin for 5 to 7 days. Differentiated C2C12 myotubes were treated with 200 nM Rot, 100 μM 3-NP, 1 mM KCN, 10 μM AMA, and 20 μM Oligo at the indicated time.

### Lentiviral-induced Nur77 KD in C2C12 myocytes

To achieve knockdown of Nur77 (Nur77^KD^) in myocytes, lentiviral particles (Sigma-Aldrich, TRCN0000234021) carrying shRNA targeted to mouse *Nr4a1* (NM_001411253) were transduced on C2C12 myotubes. Cells were harvested after two rounds (4 days) of lentiviral treatment and analyzed for KD efficiency by quantitative reverse transcription PCR and immunoblotting.

### Immunoblot analysis

Immediately snap-frozen mouse tissue samples were homogenized in RIPA lysis buffer containing freshly added protease (cOmplete EDTA-free, Roche, #0493132001) and phosphatase inhibitors (Sigma-Aldrich, P5726). All lysates were clarified by centrifugation, and protein concentrations were determined using the Pierce BCA Protein Assay Kit (Thermo Fisher Scientific, #23250). Approximately 40 μg of the total protein per sample was separated by SDS–polyacrylamide gel electrophoresis. After transfer, polyvinylidene difluoride (PVDF) membranes (MilliporeSigma, IPVH07850) were cut in half, blocked by 3% BSA (Thermo Fisher Scientific, #37520), and subsequently probed with the listed antibodies (table S1) and then imaged separately using a Bio-Rad ChemiDoc XRS imaging system. The exposure time was adjusted, and the densitometric analysis was performed using the Bio-Rad Quantity One or ImageLab software (Bio-Rad). Phosphoprotein abundance is normalized to its total protein level as indicated in the graph. The cropped images were organized to form a figure in the GraphPad Prism10 software. Mono-poly-ubiquitin antibody (ENZ-ABS840, clone UBCJ2) recognizes mono- and poly-ubiquitinylated protein conjugates. It detects both K48- and K63-linked polyubiquitin chains.

### Quantitative reverse transcription PCR

Tissues were first homogenized using the TRIzol reagent (Invitrogen), and RNA was isolated and further cleaned using RNeasy columns (Qiagen) with DNase digestion. The RNeasy Plus Kit was used for RNA isolation from cells as per the manufacturer’s instructions. cDNA synthesis was performed using 1 mg of RNA with the SuperScript II Reverse Transcriptase (Invitrogen). PCRs were prepared using the PowerUP SYBR Green Master Mix (Thermo Fisher Scientific). All PCRs were performed using QuantStudio 5 (Invitrogen). Quantification of a given gene, expressed as relative mRNA level compared with control, was calculated after normalization to a standard housekeeping gene (18*S* or Ppia). We performed separate control experiments to ensure that the efficiencies of target and reference amplification were equal, as described in the User Bulletin 2 from Applied Biosystems. Primer pairs (table S2) were designed using the Primer Express 2.0 software (Applied Biosystems) or previously published sequences. Primer sets were selected spanning at least one exon-exon junction when possible and were checked for specificity using BLAST (Basic Local Alignment Search Tool; National Center for Biotechnology Information). The specificity of the PCR amplification was confirmed by melting curve analysis, ensuring that a single product with its characteristic melting temperature was obtained.

### Electron microscopy

Muscles were harvested and immediately immersed in 2% glutaraldehyde in PBS for 2 hours at room temperature and then at 4°C overnight (*19*). Fixed tissues were washed and postfixed in a solution of 1% OsO_4_ for 2 hours. After further washes in buffer, tissues were dehydrated through serial immersions in graded ethanol solutions (50 to 100%), passed through propylene oxide, and infiltrated in mixtures of Epon 812 and propylene oxide (1:1 and then 2:1) for 2 hours each and then in pure Epon 812 overnight. Embedding was then performed in pure Epon 812, and curing was done at 60°C for 48 hours. Longitudinal sections of muscle, 60-nm thickness, were cut using an ultramicrotome (RMC MTX). Sections were double-stained in aqueous solutions of 8% uranyl acetate (25 min at 60°C) and lead citrate (3 min at room temperature). Thin sections were subsequently examined with a 100CX JEOL electron microscope.

### RNA sequencing

Frozen muscle samples were homogenized using a tissue homogenizer in TRIzol (*19*). RNA was isolated using the Qiagen RNeasy Mini QIAcube Kit following the manufacturer’s instructions. The concentration and purity of isolated RNA were determined using NanoDrop (Invitrogen) and Agilent TapeStation separately. Only samples with RNA integrity number (RIN) > 7.0 were used. Libraries were prepared using the kits of the TruSeq Stranded Total RNA Library Prep and RiboZero Gold following the manufacturer’s instructions. The resulting libraries were combined into two pools and sequenced on an Illumina Sequencing by Synthesis HiSeq 4000 analyzer within the UCLA Neuroscience Genomics Core facility. Quality of raw reads was examined using FastQC, aligned to the *Mus musculus* GRCm38, and counted using *M. musculus* GRCm38 version 97. Alignment and counting occurred using Rsubread v 2.4.2. Raw counts were then analyzed for differential gene expression using the DESeq2 v1.30.0.

### Mitochondrial proteomics

Mitochondrial pellets were lysed by resuspension in 8 M urea in 100 mM Tris (pH 8.5) and then proteolyzed with trypsin using the protein aggregation capture method (*77*). Peptide fractionation was performed on a PepSep C18 reversed-phase column (150 mm by 150 μm, 1.7-μm particle size) using an increasing gradient of acetonitrile delivered by a Thermo Fisher Scientific Vanquish Neo UHPLC. MS data were collected using a data-independent analysis (DIA) strategy on an Orbitrap Astral mass spectrometer (Thermo Fisher Scientific) (*78*). The DIA-NN algorithm was used to generate peptide and protein identifications from a human protein database downloaded from UniProt, which were then filtered using an estimated false discovery rate of less than 1% (*79*). FragPipe-Analyst was used to identify differentially expressed proteins between samples using a moderated *t* test (*80*).

### Statistics

For all animal studies, the sample size was determined using the PASS2022 software with a power of 80% and an alpha of 0.05. All cell experiments were independently repeated at least three times. Values presented are expressed as means ± SEM unless otherwise indicated. Statistical analyses were performed using Student’s *t* test when comparing two groups of samples using GraphPad Prism 10 (GraphPad Software). Significance was set a priori at *P* < 0.05.
