# Supplementary material for: Drp1 regulates mitochondrial health and controls skeletal muscle mass through the Erk1/2-Nur77 pathway
Source: Sci Adv. 2026 May 8;12(19):eaec0795. doi: 10.1126/sciadv.aec0795 (PMC13155292; doi:10.1126/sciadv.aec0795)
Supplement: Supplementary file 1 — Figs. S1 to S8 Tables S1 and S2 [file sciadv.aec0795_sm.pdf]

Science Advances
AAAS

Supplementary Materials for

Drp1 regulates mitochondrial health and controls skeletal muscle mass through the Erk1/2-Nur77 pathway

Alice M. Ma *et al.*

Corresponding author: Zhenqi Zhou, zhenqizhou@mednet.ucla.edu

Sci. Adv. **12**, eaec0795 (2026)
DOI: 10.1126/sciadv.aec0795

This PDF file includes:

Figs. S1 to S8
Tables S1 and S2

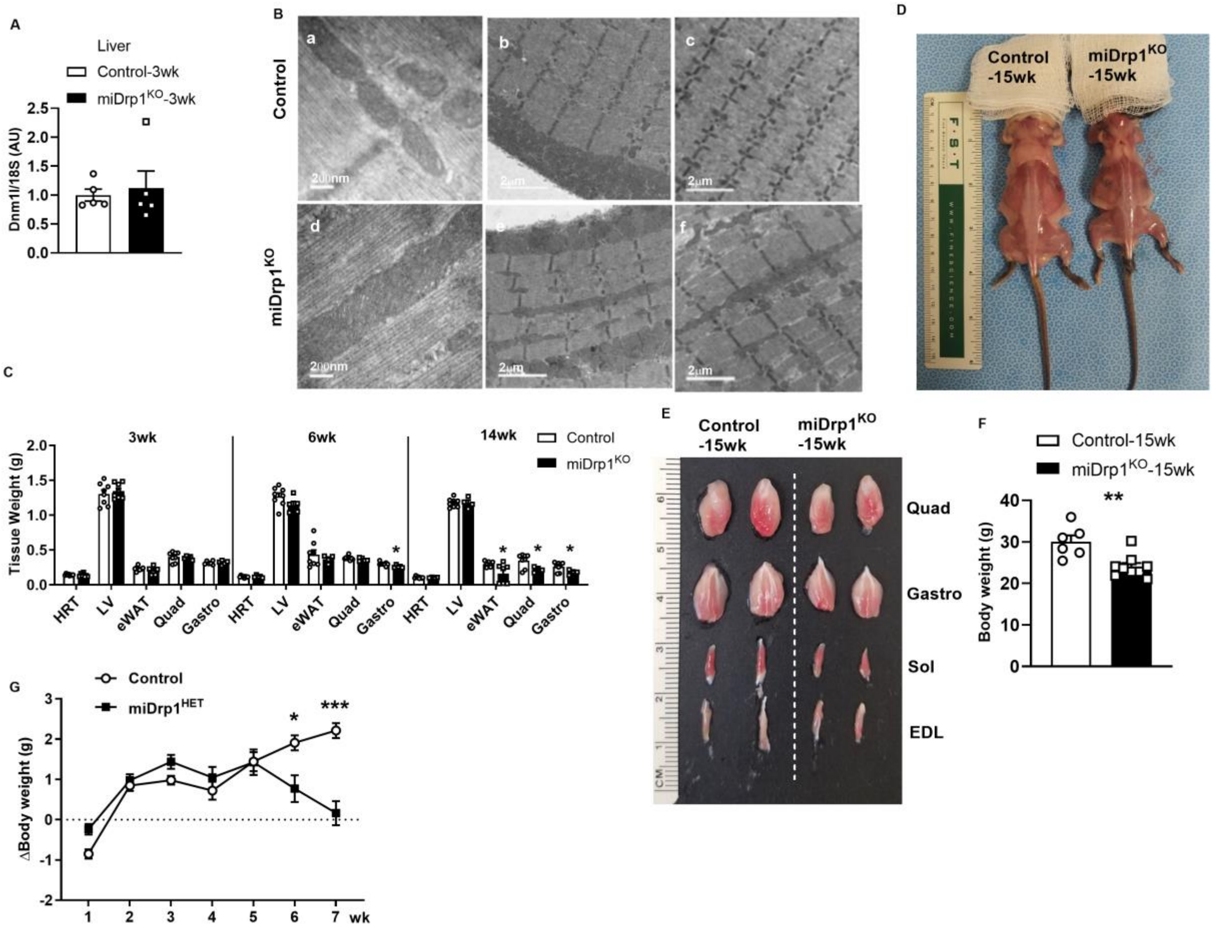

Figure S1. Acute skeletal muscle-specific *Dnm1l* deletion induces muscle wasting. A. Validation of tissue specificity of *Dnm1l* knockout. *Dnm1l* gene expression in the liver of control (n=5) and miDrp1^{KO} (n=5) mice at 3 weeks post-*Dnm1l* deletion. **B.** Electron microscopy images showing elongated mitochondrial morphology in miDrp1^{KO} mice (d, e, and f) compared to control mice (a, b, and c) at 3 weeks post-*Dnm1l* deletion. **C.** Tissue weight of male control (n=6-8) and miDrp1^{KO} mice (n=6-8) at 3, 6, and 14 weeks post-*Dnm1l* deletion. **D.** Picture of control and miDrp1^{KO} mice and **E.** muscles at 15 weeks post-*Dnm1l* deletion (single injection of TM). Quad: quadriceps, Gastro: gastrocnemius, Sol: soleus, EDL: extensor digitorum longus. **F.** Body weight of control (n=6) and miDrp1^{KO} (n=9) mice at 15 weeks post-*Dnm1l* deletion (single injection of TM). **G.** Delta body weight of control (n=6) and miDrp1^{HET} (n=6) mice. Data are presented as mean ± SEM; Unpaired student's t-test two tailed. *, $p < 0.05$; **, $p < 0.01$, ***, $p < 0.001$.

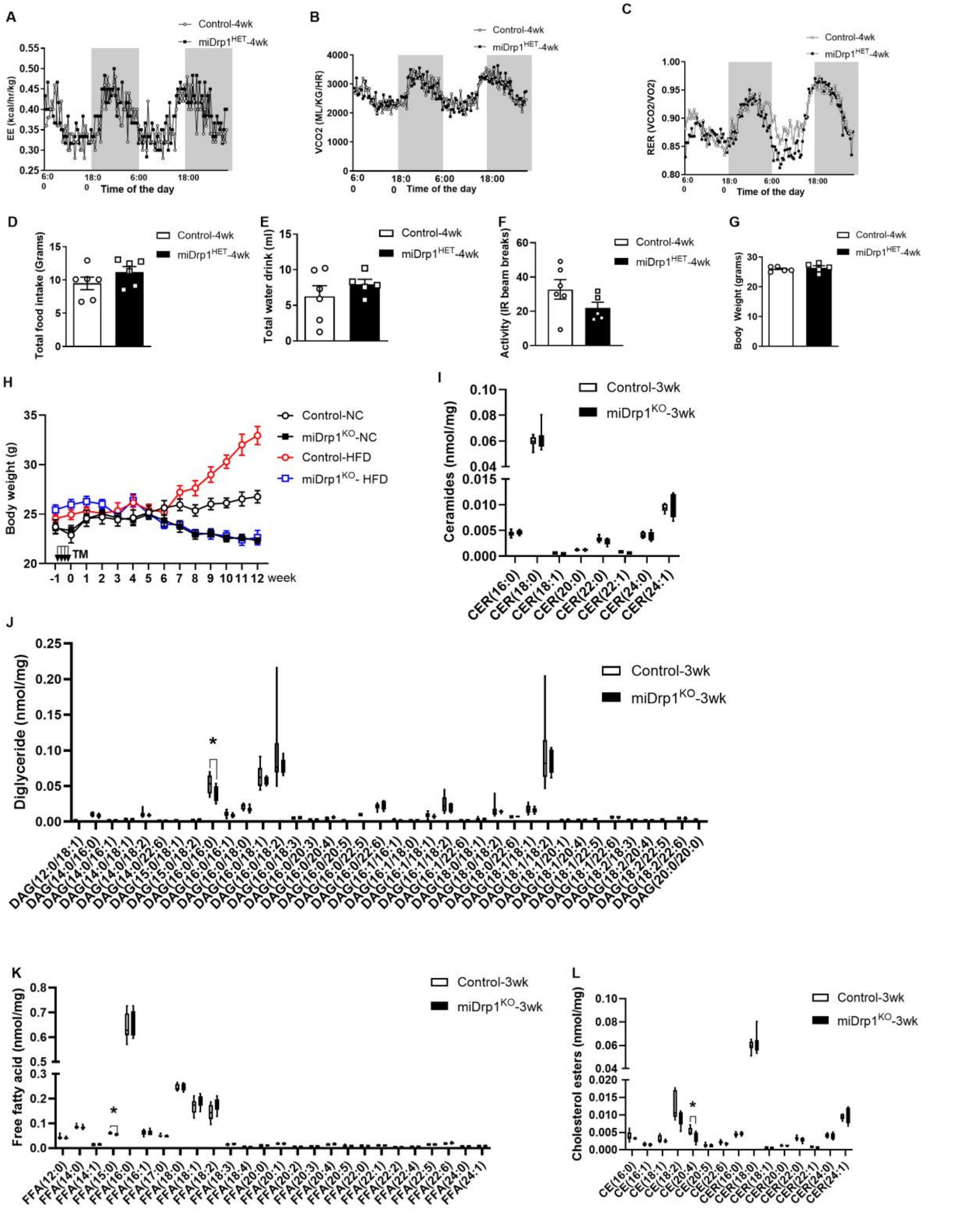

Figure S2. Comparative phenotypic analysis of conventional and inducible Drp1 deletion in skeletal muscle. A. Energy expenditure, **B.** carbon dioxide production, **C.** respiratory exchange ratio, **D.** food intake, **E.** water consumption, **F.** ambulatory activity, and **G.** body weight

of normal chow (NC)-fed control (n=6) and miDrp1^{HET} (n=6) mice at 4 weeks post-*Dnm1l* deletion. **H.** Body weight of normal chow (NC) and high-fat diet (HFD)-fed male control and miDrp1^{KO} mice (n=6). **I.** Ceramide, **J.** Diglyceride, **K.** free fatty acids, and **L.** cholesterol ester levels in gastrocnemius muscles of control (n=8) and miDrp1^{KO} (n=6) mice at 3 weeks post-*Dnm1l* deletion. Data are presented as mean ± SEM; Unpaired student's t-test two tailed. *, $p < 0.05$.

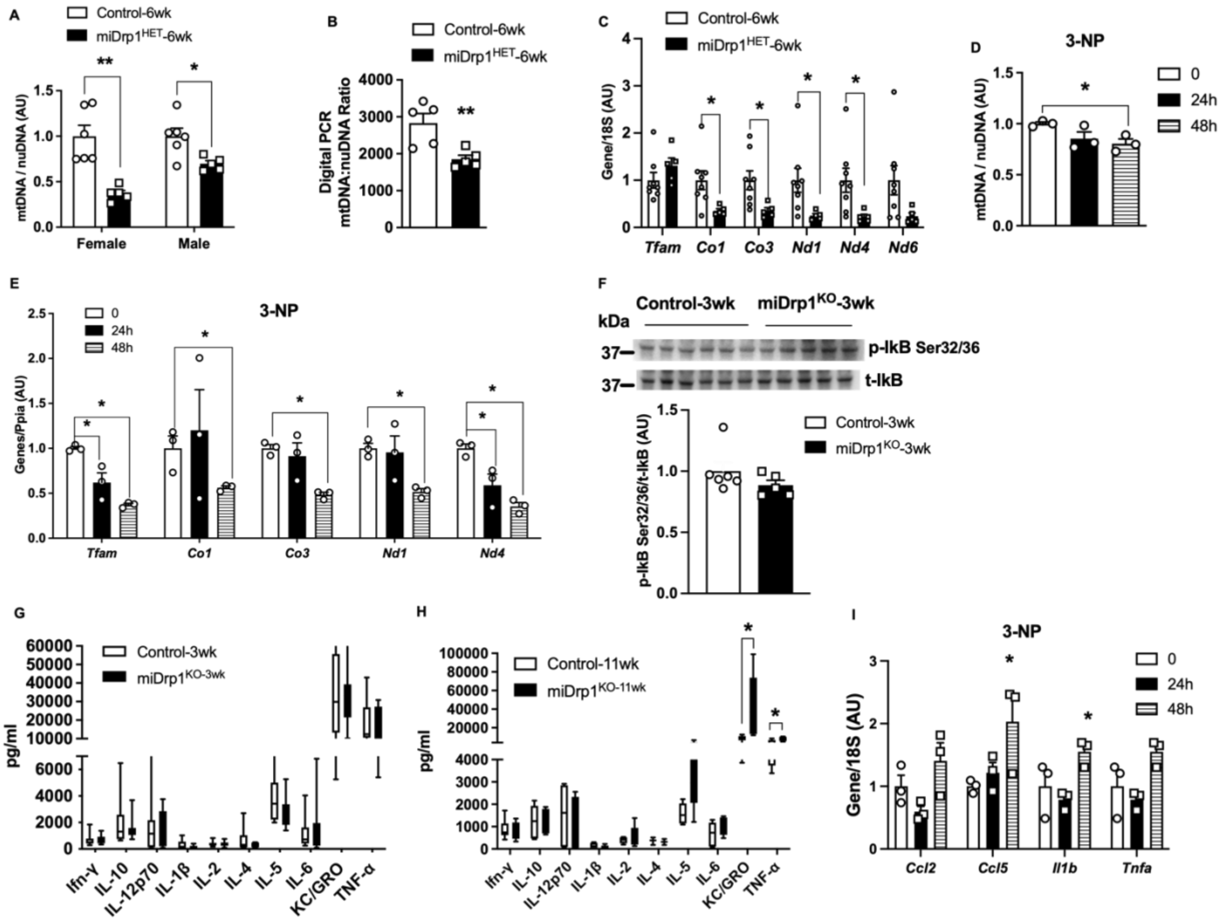

Figure S3. Acute Drp1 deletion drives progressive mtDNA depletion. A. mtDNA content in gastrocnemius muscles of both female and male control and miDrp1^{HET} mice (female: control, n=6; miDrp1^{KO}, n=5; male: control, n=6; miDrp1^{HET}, n=5) at 6 weeks post-*Dnm1l* deletion. **B.** mtDNA content in gastrocnemius muscles of female control (n=5) and miDrp1^{HET} (n=5) mice at 6 weeks post-*Dnm1l* deletion determined by digital PCR. **C.** mtDNA-encoded gene expression in gastrocnemius muscles of male control (n=8) and miDrp1^{HET} (n=5) mice at 6 weeks post-*Dnm1l* deletion. **D.** mtDNA content and **E.** mtDNA-encoded gene expression in C2C12 myotubes with 3-NP administration at indicated time (n=3). **F.** Western blot and the densitometric analysis (bottom) of phospho-IkB Ser32/36 and IkB in quadriceps muscles of controls (n=6) and miDrp1^{KO} mice (n=5) at 3 weeks post-*Dnm1l* deletion. **G.** Plasma inflammatory cytokine and chemokine levels in male control (n=11) and miDrp1^{KO} (n=11) mice at 3 weeks post-*Dnm1l* deletion. **H.** The same measurements in control (n=6) and miDrp1^{KO}, (n=6) at 11 weeks post-*Dnm1l* deletion. **I.** Inflammation gene expression was elevated after 3-NP administration in C2C12 myotubes at indicated time points (n=3). Data are presented as mean ± SEM; Unpaired student's t-test two tailed. *, $p < 0.05$.

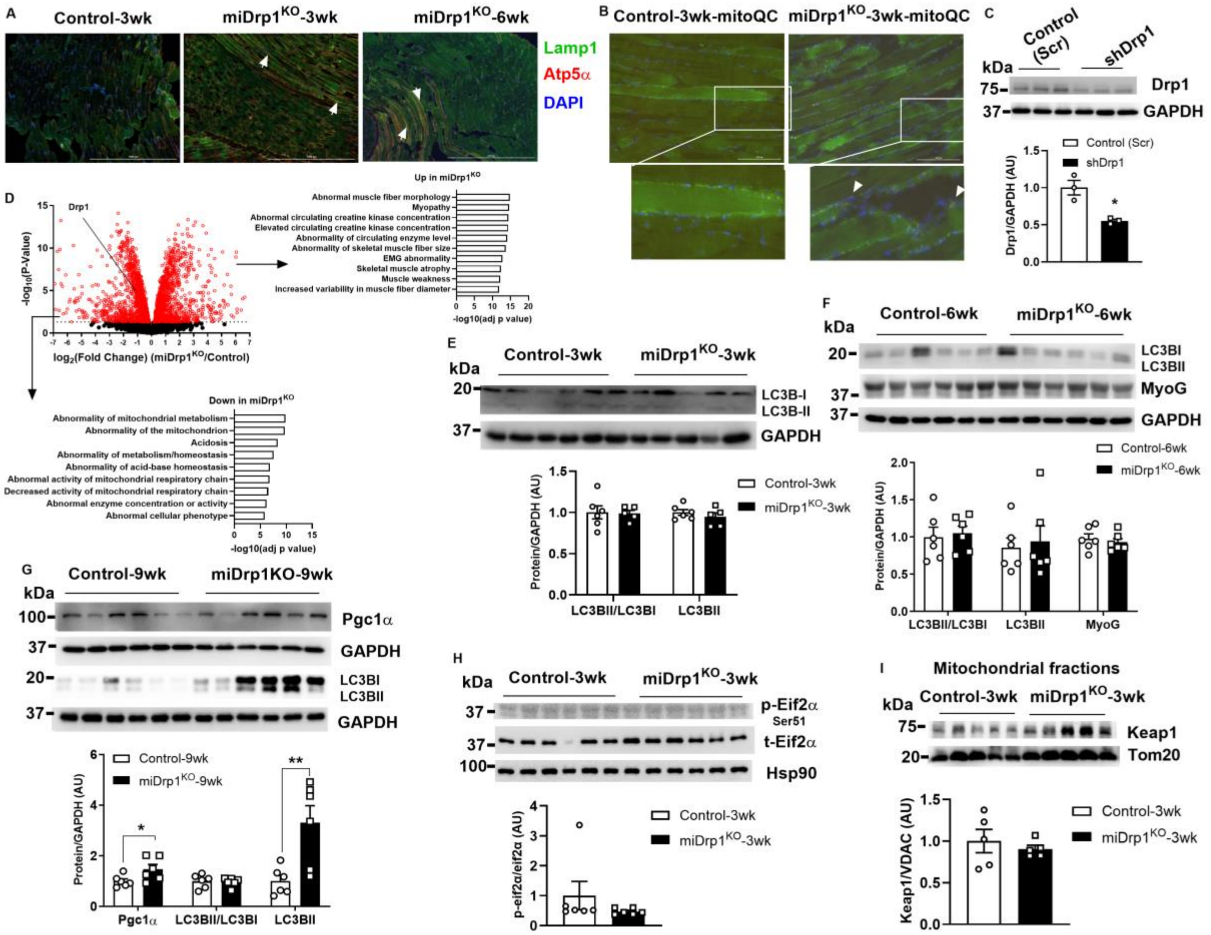

Figure S4. Drp1 deletion from skeletal muscle promotes early activation of mitochondrial degradation. A. Immunofluorescence analysis of lysosomal marker Lamp1 and mitochondrial marker Atp5α in gastrocnemius muscles of male control and miDrp1^{KO} mice at 3 and 6 weeks post-*Dnm1l* deletion (scale bar=1000μm). **B.** Immunofluorescence analysis of mitoQC in gastrocnemius muscles of male control and miDrp1^{KO} mice at 3 weeks post-*Dnm1l* deletion (scale bar=200μm). **C.** Western blot and the densitometric analysis (bottom) of Drp1 in C2C12 myotubes transduced with shDrp1 for 4 days (n=3). **D.** Volcano plot of mitochondrial proteomics performed on gastrocnemius muscles from controls (n=6) and miDrp1^{KO} (n=6) mice at 3 weeks post-*Dnm1l* deletion. Human Phenotype Ontology enrichment analysis of up (right) and down-regulated protein levels (bottom) in the isolated mitochondrial fractions from controls (n=6) and miDrp1^{KO} (n=6) mice. **E.** Western blot and the densitometric analysis (bottom) of LC3B in quadriceps muscles of controls (n=6) and miDrp1^{KO} (n=5) mice at 3 weeks post-*Dnm1l* deletion. **F.** Western blot and the densitometric analysis (bottom) of LC3B and MyoG in quadriceps muscles of controls (n=6) and miDrp1^{KO} (n=6) mice at 6 weeks post-*Dnm1l* deletion. **G.** Western blot and the densitometric analysis (bottom) of Pgc1α, LC3B in quadriceps muscles of controls (n=6) and miDrp1^{KO} (n=6) mice at 9 weeks post-*Dnm1l* deletion. **H.** Western blot and the densitometric analysis (bottom) of phosph-Eif2α Ser51 and Eif2α in quadriceps muscles of controls (n=6) and miDrp1^{KO} (n=6) mice at 3 weeks post-*Dnm1l* deletion. **I.** Western blot and the densitometric analysis (bottom) of Keap1 in mitochondrial fractions from gastrocnemius muscles of male control (n=5) and miDrp1^{KO} (n=5) mice at 3 weeks post-*Dnm1l* deletion. Data are presented as mean $\pm$ SEM; Unpaired student's t-test two tailed. *, $p < 0.05$.

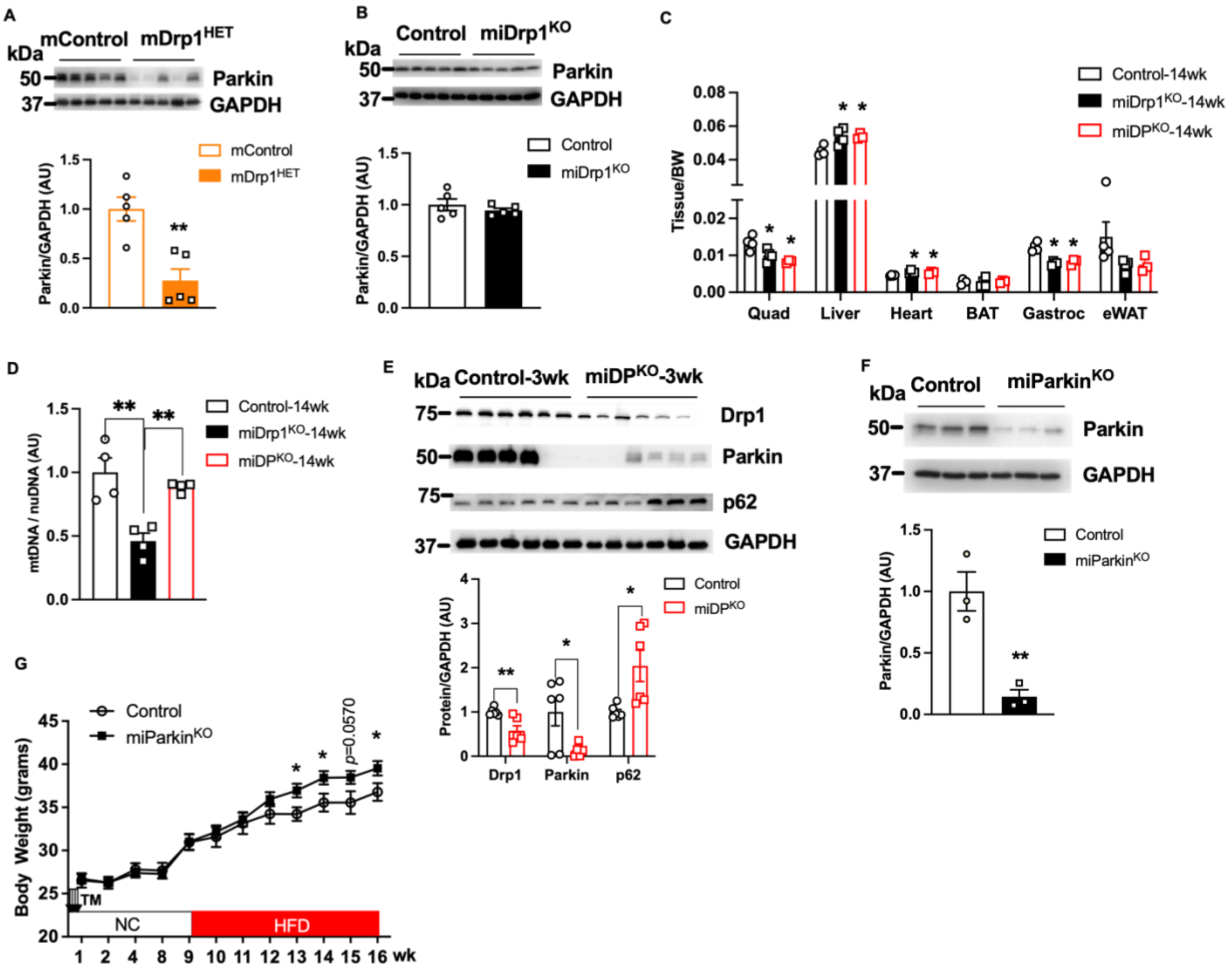

Figure S5. Parkin is required for mitochondrial degradation but not for muscle wasting in Drp1-deficient skeletal muscle. A. Western blot and the densitometric analysis (bottom) of Parkin in quadriceps muscles of mControl (n=5) and mDrp1^{HET} (n=5) mice, and **B.** Control (n=5) and miDrp1^{KO} mice (n=5) at 3 weeks post-*Dnm1l* deletion. **C.** Tissue weight of controls (n=4), miDrp1^{KO} (n=4), and miDP^{KO} (n=3) mice. **D.** mtDNA content measured by qPCR of controls (n=4), miDrp1^{KO} (n=4), and miDP^{KO} (n=4) mice. **E.** Western blot and the densitometric analysis (bottom) of Drp1, Parkin, and p62 in quadriceps muscles of controls (n=6) and miDP^{KO} (n=6) mice. **F.** Western blot and the densitometric analysis (bottom) of Parkin in quadriceps muscles of controls (n=3) and miParkin^{KO} (n=3) mice. **G.** Body weight of controls (n=7) and miParkin^{KO} (n=7) mice pre- and post-TM injection with NC and HFD feeding. Data are presented as mean ± SEM; Unpaired student's t-test two tailed. *, $p < 0.05$; **, $p < 0.01$.

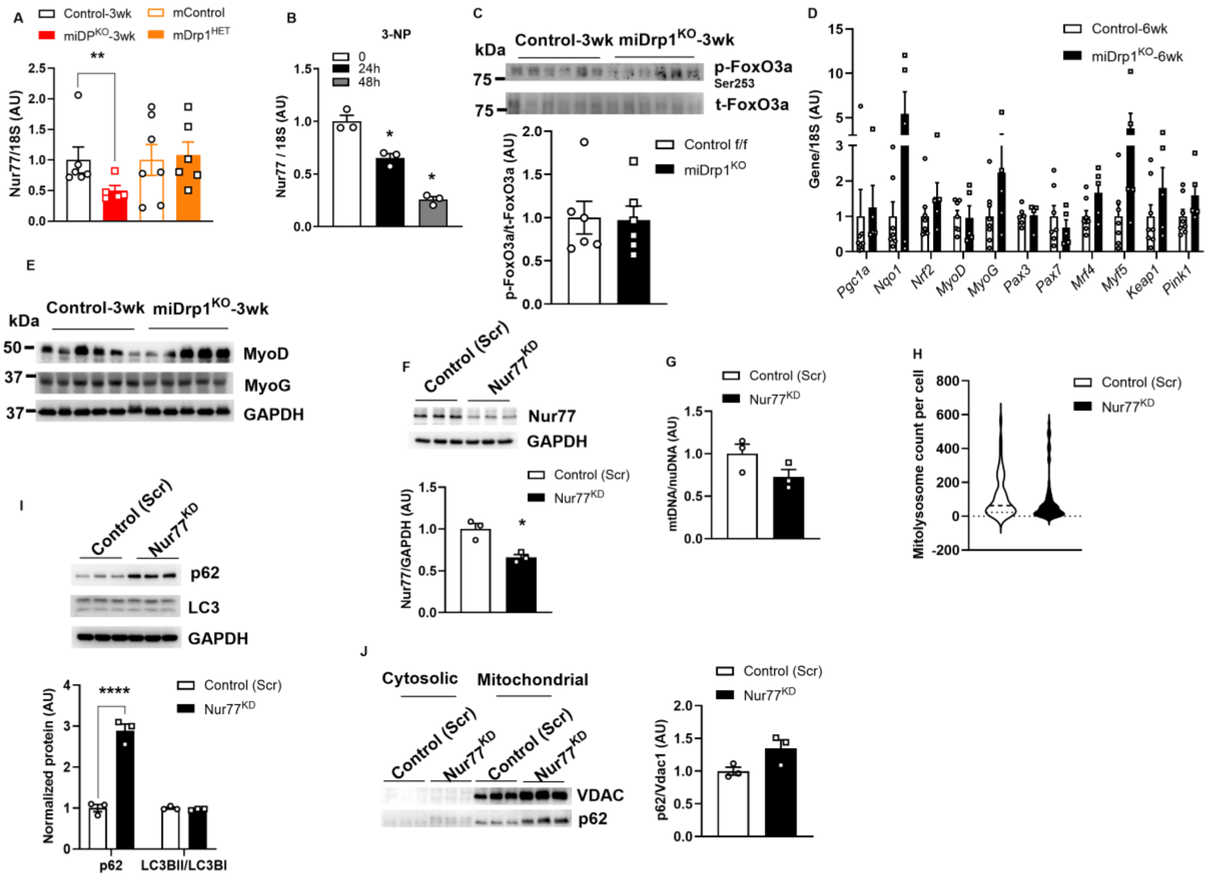

Figure S6. Acute Drp1 deletion suppresses Nur77 expression and promotes muscle atrophy. A. *Nur77* gene expression in quadriceps muscles of miDP^{KO} mice (n=5) compared to control mice (n=6) at 3 weeks post-*Dnm1l* deletion. **B.** *Nur77* gene expression in C2C12 myotubes treated with 3-NP at indicated time (n=3). **C.** Western blot and the densitometric analysis (bottom) of phospho-FoxO3a Ser253 and FoxO3a in quadriceps muscles of controls (n=6) and miDrp1^{KO} (n=6) mice at 3 weeks post-*Dnm1l* deletion. **D.** Myogenesis gene expression in quadriceps muscles of controls (n=8) and miDrp1^{KO} (n=5) mice at 6 weeks post-*Dnm1l* deletion. **E.** Western blot of MyoD and MyoG in quadriceps muscles of the second cohort of controls (n=6) and miDrp1^{KO} (n=5) mice at 3 weeks post-*Dnm1l* deletion. **F.** Western blot of Nur77 in Control (Scr) and Nur77^{KD} C2C12 myocytes (n=3). **G.** mtDNA content in Control (Scr) and Nur77^{KD} C2C12 myocytes (n=3). **H.** Mitolysosome numbers per cell in Control (Scr) (n=49) and Nur77^{KD} (n=66) C2C12 myotubes. **I.** Western blot of p62 and LC3B in Control (Scr) and Nur77^{KD} C2C12 myocytes (n=3). **J.** Western blot of p62 in the cytosolic and mitochondrial fractions of Control (Scr) and Nur77^{KD} C2C12 myocytes (n=3). Data are presented as mean ± SEM; Unpaired student's t-test two tailed. *, $p < 0.05$.

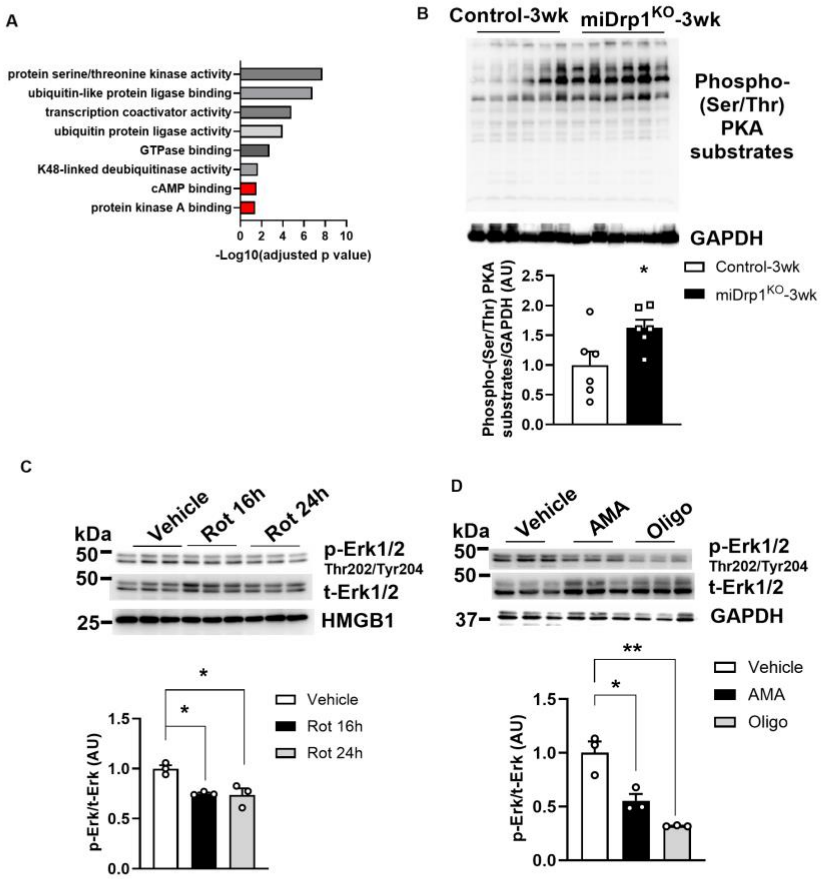

Figure S7. Acute Drp1 deletion disrupts mitochondrial function and Erk1/2-Nur77 signaling in skeletal muscle. A. Human Phenotype Ontology enrichment analysis of transcripts reduced in quadriceps muscles of miDrp1^{KO} (n=3) mice compared to control (n=3) mice. **B.** Western blot and densitometric analysis (bottom) showing increased phospho-PKA substrate protein levels in quadriceps muscles of controls (n=6) and miDrp1^{KO} (n=6) mice at 3 weeks post-*Dnm1l* deletion. **C.** Western blot of phosphorylated Erk1/2 Thr202/Tyr204 and Erk1/2 protein levels in C2C12 myotubes treated with Rot (complex I inhibitor), **D.** Antimycin A (complex III inhibitor, 16h) and Oligomycin A (complex V inhibitor, 16h) (n=3). Data are presented as mean ± SEM; Unpaired student's t-test two tailed. *, $p < 0.05$; **, $p < 0.01$.

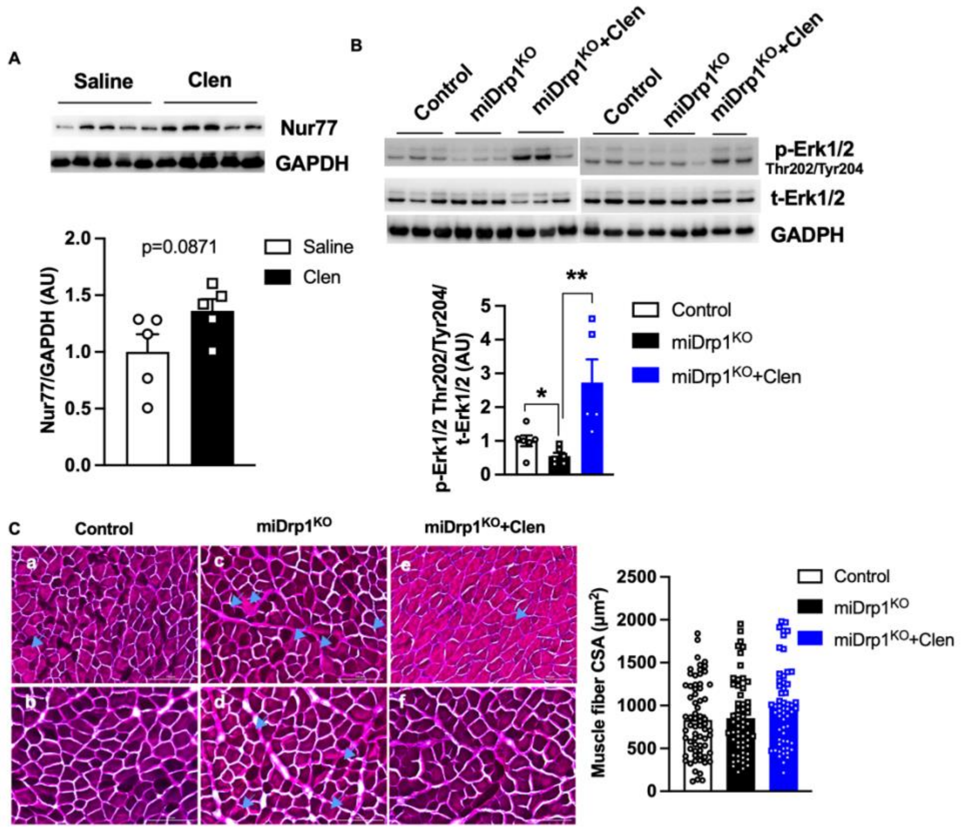

Figure S8. Clenbuterol mitigates muscle atrophy in miDrp1^{KO} mice. A. Western blot and densitometric analysis (bottom) of Nur77 in gastrocnemius muscles of WT mice treated with saline (n=5) and Clen (n=5). **B.** Western blot and densitometric analysis (bottom) of protein levels of phosphorylated Erk1/2 Thr202/Tye204 in quadriceps muscles of Control (n=6), miDrp1^{KO} (n=6), miDrp1^{KO}+Clen (n=5). **C.** At 8 weeks post-*Dnm1l* deletion, only the miDrp1^{KO}+Clen group received Clen in the drinking water for one week. H&E staining and muscle CSA (right) of gastrocnemius muscles from control (a and b), miDrp1^{KO} (c and d), and miDrp1^{KO}+Clen (e and f) mice. (Blue arrows indicate fibers with centralized nuclei, scale bar=200 μm). Data are presented as mean ± SEM; Unpaired student's t-test two tailed. *, $p < 0.05$; **, $p < 0.01$.

Table S1. Antibody list.

Antibody	Company	Catalog Number	RRID
14-3-3	Santa Cruz Biotechnology	SC-1657	AB_626618
Actin	Abcam	ab8227	AB_2305186
AlexaFluor 488 goat anti rabbit IgG	Invitrogen	A11008	AB_143165
AlexaFluor 568 goat anti mouse IgG	Invitrogen	A11004	AB_2534072
Atp5α	Abcam	ab14748	AB_301447
CamKII	Santa Cruz Biotechnology	SC-9035	AB_634551
Chop	Cell Signaling	2895T	AB_2089254
Drp1	Abcam	ab56788	AB_941306
Drp1	Cell Signaling	8570S	AB_10950498
FoxO1	Cell Signaling	2880T	AB_2106495
FoxO3a	Santa Cruz Biotechnology	SC-48348	AB_627634
Gapdh	Cell Signaling	5174S	AB_10622025
GST	Abcam	ab19256	AB_444809
HMGB1	Abcam	ab18256	AB_444360
IkB	Cell Signaling	4814T	AB_390781
Lamp1	SIGMA	L1418	AB_477157
LC3B	Cell Signaling	3868P	AB_2137707
LC3B	Cell Signaling	2775S	AB_915950
LC3B	NOVUS	NB100-2220	AB_10003146
MEK1/2	Santa Cruz Biotechnology	SC-436	AB_2142929
Mono poly-ubi	ENZO life SCIENCES	ENZ-ABS840, Clone UBCJ2	AB_2935893
mtNd1	Abcam	ab181848	AB_2687504
MyoD	Santa Cruz Biotechnology	SC-32758	AB_627978
MyoG	Abcam	ab1835	AB_302633
Nur77	Abcam	ab109180	AB_10861258
p-CamKII	Cell Signaling	3361S	AB_10015209
p-FoxO1	CST	9461S	AB_329831
p-FoxO3a	Cell Signaling	9466S	AB_2106674
p-IkB	Cell Signaling	9246S	AB_2267145
p-MEK1/2	Santa Cruz Biotechnology	SC-7995-R	AB_653313

p-p44/42	Cell Signaling	4370P	AB_2315112
p-PKA substrate	Cell Signaling	9624S	AB_331817
p-PKA C (Thr197)	Cell Signaling	5661S	AB_10707163
PKA C-α	Cell Signaling	5842S	AB_10706172
P44/42	Cell Signaling	4695	AB_390779
P62	Progen Biotechnik GmbH	03-GP62-C	AB_1542690
Parkin	Abcam	ab77924	AB_1566559
Parkin	Cell Signaling	2132	AB_10693040
Pgc1α	EMD Millipore	AB3242	AB_2268462
Pgc1α	Abcam	ab191838	AB_2721267
Sdha	Cell Signaling	11998S	AB_2750900
TFAM	Abcam	ab131607	AB_11154693
VDAC	Cell Signaling	4661S	AB_10557420

Table S2. Primer list (Mouse).

Gene	Sequence-Forward	Sequence-Reverse
18S	CGCCGCTAGAGGTGAAATTCT	CGAACCTCCGACTTTCGTTCT
Atrogin-1	GCAAACACTGCCACATTCTCT	CTTGAGGGGAAAGTGAGACG
Ccl2	AGG TCC CTG TCA TGC TTC TG	TCT GGA CCC ATT CCT TCT TG
Ccl5	CTG CTG CTT TGC CTA CCT CT	TCC TTC GAG TGA CAA ACA CG
Ccl5	GCT GCT TTG CCT ACC TCT CC	TCG AGT GAC AAA CAC GAC TGC
Dnm1l	CGTGGACTAGCTGCAGAATG	TGCCTCAGATCGTCGTAGTG
F4/80	TTTGGCTATGGGCTTCCAGTC	GCAAGGAGGACAGAGTTTATCGTG
Il1b	GACGGCACACCCACCCT	AAACCGTTTTTCCATCTTCTTT
Il6	CCACGGCCTTCCCTACTTCA	TGCAAGTGCATCATCGTTGTT
Keap1	CCAGTTGAACAGTGTGGAGC	AGCATTCCACACTGTCCAGA
Mrf4	CACAGATCGTCGGAAAGCAG	GGGTTTGTAGCTGTAGGGGT
mtCo1	TCCAACTCATCCCTTGACATC	TCCTGCTATGATAGCAAACACT
mtCo3	GCAGGATTCTTCTGAGCGTTCT	GTCAGCAGCCTCCTAGATCATGT
mtNd1	GTTGGTCCATACGGCATTTT	TGGGTGTGGTATTGGTAGGG
mtNd4	GCCTGATTACTGCCACTAATA	GGTTCCCTCATCGGGTAATAA
mtNd6	ACAACTATATATTGCCGC	GATATACGACTGCTATAGCTA
Myf5	TGAGGGAACAGGTGGAGAAC	GACAGGGCTGTTACATTCAGG
MyoD	AGGCCGTGGCAGCGA	GCTGTAATCCATGCCATCA
MyoG	CAACCAGGAGGAGCGCGATCTCCG	AGGCGCTGTGGGAGTTGCATTCACT
Nrf2	CTCGCTGGAAAAAGAAGTGG	CGGTCCAGGAGTTCAGAGAG
Nur77	CTTGAGTTCGGCAAGCCTAC	CGAGGATGAGGAAGAAGACG

Park2	GGAAGCCATAGCTGGAGTTG	AAACCTGACAGAAACGCTGG
Park6	GGATGTCGTCCTGAAGGGAG	GCTTCGCTGGAGGAACCTG
Pax3	ATCGGAGCCTTCATCTGACT	GGCGGATCTAGAAAGGAAGG
Pax7	CGGGTTCTGATTCCACATCT	CGACGAGGAAGGAGACAAGA
Peo1	GCCCAGTCACCAGTTTCCTA	ACTCTGGTCATTCACCCTCG
Ppargc1α	GGAGCCGTGACCACTGACA	TGGTTTGCTGCATGGTTCTG
Polg1	TAGCTGGCTGGTCCAAGAGT	CGACGTGGAGGTCTGCTT
Polg2	CCGTTTTCCAGCGTAGTCTC	TTCTGTGTGGCCTGGCTATT
Ppia	AGCCAAATCCTTTCTCTCCAG	CACCGTGTTCTTCGACATCA
Sdha	TACTACAGCCCCAAGTCT	TGGACCCATCTTCTATGC
Tfam	AGC TTG TAA ATG AGG CTT GGA	AGA TGT CTC CGG ATC GTT TC
Tnfα	CAC AAG ATG CTG GGA CAG TGA	TCC TTG ATG GTG GTG CAT GA
Trim63	GCGAGACAGTCGCATTTCAAA	AGGCTTGGTAAACATCTCCAG
